# Multimorbidity and healthcare utilization among home care clients with dementia in Ontario, Canada: A retrospective analysis of a population-based cohort

**DOI:** 10.1371/journal.pmed.1002249

**Published:** 2017-03-07

**Authors:** Luke Mondor, Colleen J. Maxwell, David B. Hogan, Susan E. Bronskill, Andrea Gruneir, Natasha E. Lane, Walter P. Wodchis

**Affiliations:** 1 Institute for Clinical Evaluative Sciences, Toronto, Ontario, Canada; 2 Health System Performance Research Network, Toronto, Ontario, Canada; 3 School of Pharmacy, University of Waterloo, Waterloo, Ontario, Canada; 4 School of Public Health and Health Systems, University of Waterloo, Waterloo, Ontario, Canada; 5 Department of Community Health Sciences, University of Calgary, Calgary, Alberta, Canada; 6 Division of Geriatric Medicine, University of Calgary, Calgary, Alberta, Canada; 7 Institute of Health Policy, Management, and Evaluation, University of Toronto, Toronto, Ontario, Canada; 8 Department of Family Medicine, University of Alberta, Edmonton, Alberta, Canada; 9 Toronto Rehabilitation Institute, Toronto, Ontario, Canada; University of Cambridge, UNITED KINGDOM

## Abstract

**Background:**

For community-dwelling older persons with dementia, the presence of multimorbidity can create complex clinical challenges for both individuals and their physicians, and can contribute to poor outcomes. We quantified the associations between level of multimorbidity (chronic disease burden) and risk of hospitalization and risk of emergency department (ED) visit in a home care cohort with dementia and explored the role of continuity of physician care (COC) in modifying these relationships.

**Methods and findings:**

A retrospective cohort study using linked administrative and clinical data from Ontario, Canada, was conducted among 30,112 long-stay home care clients (mean age 83.0 ± 7.7 y) with dementia in 2012. Multivariable Fine–Gray regression models were used to determine associations between level of multimorbidity and 1-y risk of hospitalization and 1-y risk of ED visit, accounting for multiple competing risks (death and long-term care placement). Interaction terms were used to assess potential effect modification by COC.

Multimorbidity was highly prevalent, with 35% (*n* = 10,568) of the cohort having five or more chronic conditions. In multivariable analyses, risk of hospitalization and risk of ED visit increased monotonically with level of multimorbidity: sub-hazards were 88% greater (sub-hazard ratio [sHR] = 1.88, 95% CI: 1.72–2.05, *p <* 0.001) and 63% greater (sHR = 1.63; 95% CI: 1.51–1.77, *p <* 0.001), respectively, among those with five or more conditions, relative to those with dementia alone or with dementia and one other condition. Low (versus high) COC was associated with an increased risk of both hospitalization and ED visit in age- and sex-adjusted analyses only (sHR = 1.11, 95% CI: 1.07–1.16, *p <* 0.001, for hospitalization; sHR = 1.07, 95% CI: 1.03–1.11, *p =* 0.001, for ED visit) but did not modify associations between multimorbidity and outcomes (Wald test for interaction, *p =* 0.566 for hospitalization and *p =* 0.637 for ED visit). The main limitations of this study include use of fixed (versus time-varying) covariates and focus on all-cause rather than cause-specific hospitalizations and ED visits, which could potentially inform interventions.

**Conclusions:**

Older adults with dementia and multimorbidity pose a particular challenge for health systems. Findings from this study highlight the need to reshape models of care for this complex population, and to further investigate health system and other factors that may modify patients’ risk of health outcomes.

## Abstract 2

In a retrospective analysis of routinely collected data, Luke Mondor and colleagues examine the associations between multimorbidity and healthcare utilization among home care clients with dementia in Ontario, Canada.

## Author summary

**Why was this study done?:**

- The co-occurrence of multiple chronic conditions in an individual (multimorbidity) has been linked to poor outcomes including increased hospital use, longer length of stays, and worse cognitive and physical functioning.
- Particularly for community-residing older adults with dementia, multimorbidity can result in challenges to both self-care and provided care. Individuals with multimorbidity often receive care from multiple physicians across different care settings each year. This lack of physician continuity may lead to poorer quality of care and outcomes.
- Important gaps exist in our understanding of the interplay between multimorbidity, health system use, and continuity of physician care specifically for individuals with dementia.
- Our historical cohort study was designed to estimate the risk of acute care hospitalization and emergency department (ED) visit by level of multimorbidity (i.e., chronic disease burden) among persons with dementia in the community. We were especially interested in whether the risk of these health outcomes was lower for those with better continuity of physician care.

**What did the researchers do and find?:**

- We performed a retrospective cohort study of 30,112 home care clients with dementia in Ontario, Canada, using routinely collected health and clinical information linked at the individual level.
- We defined the level of multimorbidity (i.e., chronic disease burden in addition to dementia diagnosis) based on a count of the presence of 16 common chronic conditions, and compared time, in days, from a health assessment to initial hospitalization (for any cause) and ED visit (not resulting in an inpatient stay) in persons with different multimorbidity levels. We accounted for other possible outcomes including death or placement in a long-term care facility.
- We found that multimorbidity was highly prevalent in this population—89% of the cohort had been diagnosed with two or more conditions in addition to dementia.
- In multivariable analyses, we found that the risk of hospitalization and ED visit increased with each higher level of multimorbidity.
- These associations were comparable in clients with dementia who had high and low degrees of physician continuity. In other words, continuity of physician care did not modify the association between level of multimorbidity and the outcomes.

**What do these findings mean?:**

- Multimorbidity is the norm rather than the exception among older adults with dementia in the home care sector. This increased chronic disease burden is associated with a greater likelihood for costly hospital admissions and emergency visits.
- With increases in life expectancy, improvements to disease detection, and a shift to community-based care, use of home care services and the prevalence of multimorbidity among older persons with dementia will likely rise. Data from this study may be useful in identifying at-risk individuals and prioritizing the deployment of limited healthcare resources.

## Introduction

Dementia (including Alzheimer disease) is a progressively debilitating condition associated with cognitive and functional impairment and behavioral challenges. As a condition affecting primarily older adults [1], most individuals with dementia also have other coexisting chronic conditions, or multimorbidity. This creates complex challenges for clinical care [2]. For example, certain conditions, such as stroke [3] and diabetes [4], have been linked to accelerated cognitive decline. Dementia-related impairments can also hinder a patient’s ability to self-manage concurrent diseases, adhere to therapies, or effectively communicate the signs and symptoms of complications to care providers, which may lead to adverse outcomes [5]. Therefore, common goals for dementia care are to manage coexisting conditions and, where possible, to prevent potentially avoidable care transitions, including hospitalization and institutionalization [6].

Healthcare utilization, including hospital admissions [7–11] and emergency department (ED) visits [10–13], has been shown to be elevated among older adults diagnosed with dementia. Hospital and ED visits are particularly relevant for persons with dementia given their heightened risks for cognitive and functional decline during and after hospitalization [14–16]. Such vulnerability places these patients at further risk of additional care transitions following acute care discharge, including readmission to the ED [12] or hospital or placement in a long-term care (LTC) facility [17], as well as death [18]. Associations between these outcomes and multimorbidity are poorly understood within the dementia population, but may be useful for healthcare providers and policymakers in targeting high-risk individuals for enhanced patient-centered care, and improving patient integration across all sectors of the healthcare system.

A further concern is that older adults with dementia and coexisting conditions may be susceptible to care fragmentation, as they frequently receive care from multiple physicians across the healthcare system each year [19]. This can lead to deficiencies in care delivery, including poor communication between providers and medication errors. A recent US study of older adults with dementia found that lower continuity of physician care (COC) was associated with greater healthcare utilization [20], but important gaps exist in our understanding of utilization patterns among persons with dementia and multimorbidity, particularly in the context of healthcare systems that provide universal access to services.

In the current study, we used linked population-based health administrative and clinical assessment data to quantify associations between multimorbidity and two healthcare utilization outcomes, hospital admissions and ED visits, among community-residing individuals with dementia in Ontario, Canada. We focused specifically on individuals with dementia in the home care setting. In Ontario, as elsewhere, the home care sector is a growing component of healthcare delivery that helps older adults to maintain independence within their home residence while reducing overall health system costs. Individuals with dementia comprise a large proportion of this population [21,22]. As a secondary objective, we tested whether COC had a direct association with healthcare utilization and whether COC modified the associations between multimorbidity and the hospitalization and ED visit outcomes. In a recently published study [23], the association between multimorbidity and hospitalization was less pronounced among individuals with greater physician continuity in the general population. We hypothesized that similar findings of effect modification by COC would be observed among persons with dementia.

## Methods

This study was approved by the Research Ethics Board of Sunnybrook Health Sciences Centre (Toronto, Canada). As we used health information routinely collected in Ontario, informed consent from study participants was not required. The study is reported per RECORD guidelines (S1 Text). The study protocol is available in S2 Text.

### Study design and setting

We conducted a retrospective analysis of linked population-based health administrative and clinical assessment data in Ontario, Canada’s largest province and home to over 13 million residents. Almost all Ontarians are covered by a universal health insurance program that pays for all medically necessary inpatient, emergency, and physician services, and includes coverage for medications for individuals 65 y of age and older. Publicly funded home care services and LTC placements are coordinated and delivered through regional Community Care Access Centres. Home care services are provided on a short- or long-stay basis. The latter refers to ongoing supportive care required for more than 60 d in a single episode. In Ontario, it is mandatory for all long-stay home care clients to be assessed with a comprehensive clinical assessment tool, the Resident Assessment Instrument for Home Care (RAI-HC), on a semiannual basis.

All services provided via the public health insurance system are recorded in databases and later linked deterministically using unique encoded identifiers and analyzed at the Institute for Clinical Evaluative Sciences in Toronto. Each dataset used in this study is described in S1 Table.

### Study population

We identified all Ontario residents aged 50 y and older who received a RAI-HC assessment between January 1, 2012, and June 30, 2012, and had been diagnosed with dementia prior to the assessment date. Dementia was identified based on the presence of (1) a relevant diagnostic code (S2 Table) recorded on a hospital discharge, (2) a relevant diagnostic code recorded on three physician billings separated by at least 30 d occurring within a 2-y period, or (3) dispensing of any cholinesterase inhibitor (whose only indications are for the treatment of Alzheimer disease or dementia associated with Parkinson disease). This case ascertainment algorithm has been validated in Ontario [24]. We restricted our population to individuals with a RAI-HC assessment in order to include only those with dementia living in the community and to obtain additional health and functional status information for the study population that is available only from the RAI-HC. We excluded individuals if they had missing information on age or sex, or were not eligible for healthcare coverage at the time of RAI-HC assessment. One RAI-HC assessment per individual was selected for analysis (index RAI-HC assessment), the nearest to April 1, 2012. Counts according to the study exclusion criteria are shown in S3 Table.

### Exposure

For all individuals included in the study, we determined the presence of 16 comorbid conditions. Each condition was defined at the time of index RAI-HC assessment using historical data. Consistent with previous studies on multimorbidity in Ontario [22,23,25,26], these conditions were selected based on their large economic impact and high prevalence in the general population [27–29], and included the following: acute myocardial infarction, asthma, any cancer, cardiac arrhythmia, chronic coronary syndrome, chronic obstructive pulmonary disorder, congestive heart failure, diabetes, hypertension, non-psychotic mood and anxiety disorders, other mental illnesses (which included schizophrenia, delusions, and other psychoses; personality disorders; and substance abuse), osteoarthritis, osteoporosis, renal failure, rheumatoid arthritis, and stroke (excluding transient ischemic attack). All cases were identified from Ontario Health Insurance Plan database and Discharge Abstract Database (DAD) data using ICD-9 and -10 codes. Validated case ascertainment algorithms (where available) or similar case definition approaches were used (S2 Table). For these conditions, for each individual we defined level of multimorbidity (i.e., chronic disease burden) as the number of prevalent chronic conditions in addition to dementia. This was coded as 0–1, 2, 3, 4, or 5+.

### Covariates

As proxies for disease severity, we derived two independent markers from the RAI-HC data, the Minimum Data Set Health Status Index (MDS-HSI), and the Changes in Health, End-stage disease and Symptoms and Signs (CHESS) scale. The MDS-HSI is a preference-based measure of health-related quality of life that includes six domains (sensation, mobility, emotion, cognition, self-care, and pain) [30]. Values range from 1.00 (perfect health state) to −0.02 (health state worse than death, which is scored at 0). In contrast, CHESS is an ordinal measure used to detect instability in health for older adults and has been shown to be a strong predictor of hospitalization and mortality in older adults [31,32]. We coded values to range from 0 (no health instability) to 4 (high to very high instability).

We measured COC using the Bice–Boxerman continuity of care index [33]. This index measures the extent to which a patient visits the same clinician for ongoing medical care over a defined period. Values range from 0 to 1, where scores approaching 1 reflect a higher concentration of visits to a single physician. We included all ambulatory visits over the 2 y prior to the index RAI-HC assessment and considered all physician specialties because both primary care physicians and specialists play a role in the management of chronic conditions. The Bice–Boxerman index accounts for physician referrals. Each individual was categorized as having either a high COC or low COC, with high COC defined as a Bice-Boxerman index value greater than or equal to the median score in the study population. Alternative COC measures were explored in sensitivity analyses, as noted below.

Age, sex, and date of death (where applicable) were identified from the Ontario Registered Persons Database, and neighborhood-level income quintile and rurality (urban or rural residence) [34] from the 2006 census. Marital status was derived from the index RAI-HC assessment; categories included married, separated/divorced, widowed, and never married. All LTC placements following the index RAI-HC event (where applicable) were derived from Continuing Care Reporting System data. Lastly, the number of acute hospital episodes and the number of unplanned ED visits that each client experienced in the 1 y prior to their index RAI-HC assessment were identified from the DAD and National Ambulatory Care Reporting System datasets, respectively.

### Outcomes

We followed each individual prospectively for 1 y following their index RAI-HC assessment to identify the time (in days) to (1) first acute inpatient hospital admission (DAD data) and (2) first unplanned ED visit that did not result in an inpatient stay (National Ambulatory Care Reporting System data). For both measures, all causes were considered.

### Analysis

We described the distribution of baseline sociodemographic characteristics, clinical characteristics, and the frequency of prior health service utilization by level of multimorbidity. We modeled associations between level of multimorbidity and the risk of (1) acute hospitalization and (2) unplanned ED visit with a competing risks regression derived from Fine and Gray’s proportional sub-hazards model [35] using Stata’s *stcrreg* command. This regression is based on the cumulative incidence function, which quantifies the probability of an event of interest (hospitalization or ED visit) during the study follow-up period, acknowledging the possibility of one or more competing events. Deaths and placements into LTC facilities were considered competing events. Individuals who did not experience any outcome (event, death, or LTC admission) were censored at the end of the 1-y observation period. We derived age- and sex-adjusted associations between level of multimorbidity and each outcome. Multivariable competing risk regressions then assessed the risk of hospitalization or ED visit by level of multimorbidity adjusting for age, sex, income quintile, rurality, marital status, COC, health-related quality of life (MDS-HSI), CHESS, prior hospitalizations, and prior ED visits. Plots of Schoenfeld residuals were used to assess the assumption of proportionality, which was not violated in any of the models. Individuals missing information on any covariate were excluded from each analysis; however, no single covariate had more than 1.8% missing (COC).

To determine whether COC modified the association between level of multimorbidity and the hospitalization and ED visit outcomes, an interaction term was added to each multivariable model. We plotted resulting coefficients for visual representation of effect modification and used Stata’s post-estimation *lincom* command to assess statistical differences in the risk of each outcome by COC with increasing levels of multimorbidity. Ten sub-hazard ratio (sHR) estimates were derived: one per level of multimorbidity (*n* = 5) per outcome (*n* = 2). In addition, for both outcomes, a Wald test was used to assess whether the sHRs associated with low COC were the same for each level of multimorbidity.

Sensitivity analyses assessed the influence of particular subgroups expected to have either a higher or lower baseline risk of admission to hospital or to the ED. Specifically, multivariable analyses were repeated that excluded from our study population individuals whose index RAI-HC assessment was related to either a “change in status” or “review at the return from hospital” (sensitivity analysis 1) and individuals living with modest to very severe cognitive impairment, based on Cognitive Performance Scale value ≥ 4 (sensitivity analysis 2). We also assessed the robustness of our COC measure and effect modification findings by recalculating COC by excluding individuals with <3 physician visits (sensitivity analysis 3) and also by categorizing COC into tertiles (low, medium, and high COC; sensitivity analysis 4).

## Results

We identified 30,112 individuals in Ontario with dementia who had a RAI-HC assessment between January 1 and June 30, 2012. They represented 27.5% of all home care clients otherwise eligible for study inclusion (S3 Table). Table 1 presents characteristics of the study population. The mean age of the study population was 83.0 (standard deviation 7.7) y, 63% were women (*n =* 19,056), and 88% lived in an urban setting (*n =* 26,461). Eleven percent of the study population (*n =* 3,309) was diagnosed with dementia alone (*n =* 755) or with dementia and one other condition (*n =* 2,554). A total of 89% (*n =* 26,804) had two or more conditions in addition to dementia, while 35% (*n =* 10,568) had five or more comorbid conditions in addition to dementia. The most prevalent comorbid conditions were hypertension (82.4%), osteoarthritis (59.7%), and diabetes (34.4%) (S4 Table). Both proxies for disease severity (MDS-HSI and CHESS) showed greater impairment with higher levels of multimorbidity, while prior healthcare utilization (hospitalizations and ED visits in the past 1 y) increased with increasing number of chronic conditions. Median COC in the population was 0.63. Continuity decreased with each additional level of multimorbidity.

**Table 1:** Profile of long-stay home care clients with dementia in Ontario in 2012, by level of multimorbidity (number of prevalent chronic conditions in addition to dementia).

Variable	Overall (*n =* 30,112)	Number of chronic conditions in addition to dementia				
		0–1 (*n =* 3,309)	2 (*n =* 4,799)	3 (*n =* 5,847)	4 (*n =* 5,589)	5+ (*n =* 10,568)
**Sociodemographics**						
Age (years), mean ± SD	83.0 ± 7.7	80.9 ± 9.2	82.8 ± 7.8	83.1 ± 7.6	83.6 ± 7.4	83.5 ± 7.1
Women	19,056 (63.3%)	2,031 (61.4%)	3,208 (66.8%)	3,839 (65.7%)	3,607 (64.5%)	6,371 (60.3%)
Urban resident	26,461 (87.9%)	2,839 (85.8%)	4,142 (86.3%)	5,112 (87.4%)	4,918 (88.0%)	9,450 (89.4%)
**Income quintile**						
1 (low)	6,359 (21.1%)	632 (19.1%)	960 (20.0%)	1,231 (21.1%)	1,197 (21.4%)	2,339 (22.1%)
2	6,095 (20.2%)	648 (19.6%)	962 (20.0%)	1,142 (19.5%)	1,164 (20.8%)	2,179 (20.6%)
3	5,786 (19.2%)	656 (19.8%)	951 (19.8%)	1,135 (19.4%)	1,059 (18.9%)	1,985 (18.8%)
4	5,919 (19.7%)	651 (19.7%)	961 (20.0%)	1,195 (20.4%)	1,078 (19.3%)	2,034 (19.2%)
5 (high)	5,832 (19.4%)	708 (21.4%)	940 (19.6%)	1,121 (19.2%)	1,067 (19.1%)	1,996 (18.9%)
**Marital status**						
Married	12,563 (41.7%)	1,487 (44.9%)	1,946 (40.6%)	2,424 (41.5%)	2,260 (40.4%)	4,446 (42.1%)
Widowed	14,439 (48.0%)	1,403 (42.4%)	2,352 (49.0%)	2,864 (49.0%)	2,778 (49.7%)	5,042 (47.7%)
Separated/divorced	1,744 (5.8%)	217 (6.6%)	258 (5.4%)	318 (5.4%)	302 (5.4%)	649 (6.1%)
Never married/other	1,366 (4.5%)	202 (6.1%)	243 (5.1%)	241 (4.1%)	249 (4.5%)	431 (4.1%)
**MDS-HSI**						
Mean ± SD	0.45 ± 0.21	0.48 ± 0.22	0.47 ± 0.21	0.46 ± 0.21	0.44 ± 0.20	0.43 ± 0.19
Median (IQR)	0.63 (0.40–0.88)	0.69 (0.40–1.00)	0.67 (0.44–1.00)	0.66 (0.41–0.90)	0.64 (0.42–0.87)	0.59 (0.38–0.82)
**CHESS scale**						
No instability	7,274 (24.2%)	944 (28.5%)	1,283 (26.7%)	1,508 (25.8%)	1,322 (23.7%)	2,217 (21.0%)
Minimal instability	9,132 (30.3%)	1,001 (30.3%)	1,475 (30.7%)	1,777 (30.4%)	1,720 (30.8%)	3,159 (29.9%)
Low instability	8,825 (29.3%)	969 (29.3%)	1,383 (28.8%)	1,721 (29.4%)	1,662 (29.7%)	3,090 (29.2%)
Moderate instability	3,658 (12.1%)	301 (9.1%)	514 (10.7%)	627 (10.7%)	654 (11.7%)	1,562 (14.8%)
High to very high instability	1,223 (4.1%)	94 (2.8%)	144 (3.0%)	214 (3.7%)	231 (4.1%)	540 (5.1%)
**Prior acute hospitalizations (past year)**						
None	18,121 (60.2%)	2,611 (78.9%)	3,468 (72.3%)	3,876 (66.3%)	3,364 (60.2%)	4,802 (45.4%)
1	7,920 (26.3%)	573 (17.3%)	1,052 (21.9%)	1,440 (24.6%)	1,563 (28.0%)	3,292 (31.2%)
2+	4,071 (13.5%)	125 (3.8%)	279 (5.8%)	531 (9.1%)	662 (11.8%)	2,474 (23.4%)
**Prior emergency department visits (past year)**						
None	15,292 (50.8%)	2,130 (64.4%)	2,804 (58.4%)	3,180 (54.4%)	2,765 (49.5%)	4,413 (41.8%)
1	7,412 (24.6%)	706 (21.3%)	1,158 (24.1%)	1,425 (24.4%)	1,408 (25.2%)	2,715 (25.7%)
2+	7,408 (24.6%)	473 (14.3%)	837 (17.4%)	1,242 (21.2%)	1,416 (25.3%)	3,440 (32.6%)
**Continuity of care (Bice-Boxerman index)**^1^						
Mean ± SD	0.63 ± 0.28	0.66 ± 0.31	0.66 ± 0.28	0.64 ± 0.28	0.63 ± 0.27	0.59 ± 0.27
Median (IQR)	0.63 (0.40–0.88)	0.69 (0.40–1.00)	0.67 (0.44–1.00)	0.66 (0.41–0.90)	0.64 (0.42–0.87)	0.59 (0.38–0.82)
Low (< median)	14,754 (49.0%)	1,420 (42.9%)	2,154 (44.9%)	2,739 (46.8%)	2,714 (48.6%)	5,727 (54.2%)
High (≥ median)	14,825 (49.2%)	1,703 (51.5%)	2,536 (52.8%)	3,010 (51.5%)	2,805 (50.2%)	4,771 (45.1%)

Data are given as number (percent) unless otherwise indicated. CHESS values to range from 0 (no health instability) to 4 (high to very high instability), with higher values indicative of adverse outcomes.

^1^Includes only study participants with one or more ambulatory visits over the 2 y prior to the index Resident Assessment Instrument for Home Care assessment (1.8% missing information).

CHESS, Changes in Health, End-stage disease and Symptoms and Signs; IQR, interquartile range; MDS-HSI, Minimum Data Set Health Status Index; SD, standard deviation.

Table 2 shows that 29% (*n =* 8,759) and 34% (*n =* 10,189) of the study population experienced an acute care hospitalization or ED visit, respectively, as their first event during the 1-y follow-up period (data presented by high and low COC in S5 Table). Both of the proportions increased with higher levels of multimorbidity, from 19% (0–1 chronic conditions) to 37% (5+ chronic conditions) for hospitalization and from 25% (0–1 chronic conditions) to 40% (5+ chronic conditions) for ED visit. Overall, 18% (*n =* 5,298) of the study population died at any point during the 1-y follow-up period, and 38% (*n =* 11,349) entered LTC.

**Table 2:** Proportion of long-stay home care clients with dementia in Ontario in 2012 who experienced each outcome (any acute hospitalization or any emergency department visit) during the 1-y follow-up period, accounting for multiple competing risks (death, long-term care admission, censoring at end of follow-up).

Outcome	Overall (*n =* 30,112)	Number of chronic conditions in addition to dementia				
		0–1 (*n =* 3,309)	2 (*n =* 4,799)	3 (*n =* 5,847)	4 (*n =* 5,589)	5+ (*n =* 10,568)
**Acute hospitalization**						
Censored	11,220 (37.3%)	1,312 (39.6%)	1,922 (40.1%)	2,329 (39.8%)	2,153 (38.5%)	3,504 (33.2%)
Event	8,759 (29.1%)	633 (19.1%)	1,083 (22.6%)	1,485 (25.4%)	1,616 (28.9%)	3,942 (37.3%)
Died	1,141 (3.8%)	128 (3.9%)	143 (3.0%)	216 (3.7%)	183 (3.3%)	471 (4.5%)
Admitted to LTC	8,992 (29.9%)	1,236 (37.4%)	1,651 (34.4%)	1,817 (31.1%)	1,637 (29.3%)	2,651 (25.1%)
**Emergency department visit**						
Censored	8,824 (29.3%)	1,069 (32.3%)	1,536 (32.0%)	1,817 (31.1%)	1,684 (30.1%)	2,718 (25.7%)
Event	10,189 (33.8%)	831 (25.1%)	1,363 (28.4%)	1,866 (31.9%)	1,954 (35.0%)	4,175 (39.5%)
Died	2,104 (7.0%)	186 (5.6%)	279 (5.8%)	385 (6.6%)	354 (6.3%)	900 (8.5%)
Admitted to LTC	8,995 (29.9%)	1,223 (37.0%)	1,621 (33.8%)	1,779 (30.4%)	1,597 (28.6%)	2,775 (26.3%)
**Any competing risk**^1^						
Died	5,298 (17.6%)	436 (13.2%)	681 (14.2%)	932 (15.9%)	945 (16.9%)	2,304 (21.8%)
Admitted to LTC	11,349 (37.7%)	1,460 (44.1%)	1,987 (41.4%)	2,225 (38.1%)	2,078 (37.2%)	3,599 (34.1%)

Data are given as number (percent).

^1^Frequencies over the 1-y follow-up period, regardless of first event.

LTC, long-term care.

Results from age- and sex-adjusted and multivariable competing risk analyses are shown in Table 3. For both hospitalization and ED visit, risk increased monotonically with higher levels of multimorbidity. All comparisons (reference group is 0–1 chronic conditions) were statistically significant (*p <* 0.05). For hospitalization, risk among those with five or more conditions was more than double (sHR = 2.18, 95% CI: 2.00–2.37) that of individuals with 0–1 conditions in age- and sex-adjusted analyses. In the full model, the sHR was reduced slightly to 1.88 (95% CI: 1.72–2.05). Similarly for ED visit, the age- and sex-adjusted risk was 76% greater among those with five or more conditions (versus 0–1 conditions, sHR = 1.76, 95% CI: 1.63–1.90). In the full model, sub-hazards were 63% greater (sHR = 1.63, 95% CI: 1.51–1.77).

**Table 3:** Association between level of multimorbidity (number of diagnosed chronic conditions) and 1-y risk of acute hospitalization and emergency department visit among long-stay home care clients with dementia in Ontario in 2012.

	Outcome: hospitalization				Outcome: ED visit			
	Age- and sex-adjusted model		Multivariable model		Age- and sex-adjusted model		Multivariable model	
	sHR (95% CI)	*p*-Value	sHR (95% CI)	*p*-Value	sHR (95% CI)	*p*-Value	sHR (95% CI)	*p*-Value
**Level of multimorbidity**								
0–1 CCs	1.00 [Ref]		1.00 [Ref]		1.00 [Ref]		1.00 [Ref]	
2 CCs	1.20 (1.09–1.33)	<0.001	1.17 (1.06–1.29)	0.003	1.17 (1.07–1.27)	0.001	1.15 (1.05–1.26)	0.002
3 CCs	1.37 (1.25–1.51)	<0.001	1.30 (1.18–1.43)	<0.001	1.34 (1.24–1.46)	<0.001	1.29 (1.18–1.40)	<0.001
4 CCs	1.60 (1.46–1.75)	<0.001	1.48 (1.34–1.63)	<0.001	1.50 (1.39–1.63)	<0.001	1.43 (1.31–1.56)	<0.001
5+ CCs	2.18 (2.00–2.37)	<0.001	1.88 (1.72–2.05)	<0.001	1.76 (1.63–1.90)	<0.001	1.63 (1.51–1.77)	<0.001
**Sex**								
Women	1.00 [Ref]		1.00 [Ref]		1.00 [Ref]		1.00 [Ref]	
Men	1.21 (1.16–1.27)	<0.001	1.18 (1.13–1.24)	<0.001	0.98 (0.95–1.03)	0.452	0.94 (0.90–0.99)	0.011
**Age (continuous)**	1.00 (1.00–1.01)	0.003	1.01 (1.00–1.01)	<0.001	0.99 (0.00–1.00)	<0.001	1.00 (1.00–1.00)	0.453
**Income quintile**								
1 (low)			1.02 (0.96–1.09)	0.466			1.07 (1.00–1.14)	0.035
2			1.01 (0.94–1.08)	0.797			1.13 (1.06–1.20)	<0.001
3			0.95 (0.89–1.02)	0.128			1.05 (0.98–1.12)	0.138
4			0.91 (0.85–0.98)	0.008			1.04 (0.97–1.11)	0.246
5 (high)			1.00 [Ref]				1.00 [Ref]	
**Marital status**								
Married			1.00 [Ref]				1.00 [Ref]	
Widowed			0.98 (0.93–1.03)	0.327			0.95 (0.90–0.99)	0.021
Separated/divorced			0.98 (0.90–1.08)	0.754			0.93 (0.86–1.02)	0.121
Never married/other			0.86 (0.77–0.97)	0.010			0.87 (0.78–0.96)	0.005
**Residence**								
Urban			1.00 [Ref]				1.00 [Ref]	
Rural			1.03 (0.96–1.10)	0.424			1.18 (1.12–1.25)	<0.001
**Continuity of care**								
Low (<median)			1.02 (0.97–1.06)	0.418			1.03 (0.99–1.07)	0.159
High (≥median)			1.00 [Ref]				1.00 [Ref]	
**Prior hospitalizations**								
None			1.00 [Ref]				1.00 [Ref]	
1			1.14 (1.09–1.20)	<0.001			0.94 (0.89–0.98)	0.010
≥2			1.40 (1.31–1.49)	<0.001			1.04 (0.98–1.11)	0.203
**Prior ED visits**								
None			1.00 [Ref]				1.00 [Ref]	
1			1.22 (1.16–1.29)	<0.001			1.36 (1.30–1.43)	<0.001
≥2			1.39 (1.32–1.47)	<0.001			1.94 (1.85–2.04)	<0.001
**MDS-HSI (continuous)**			1.25 (1.12–1.39)	<0.001			2.36 (2.13–2.62)	<0.001
**CHESS scale**								
No instability			1.00 [Ref]				1.00 [Ref]	
Minimal instability			1.08 (1.02–1.14)	0.012			1.01 (0.96–1.06)	0.709
Low instability			0.98 (0.93–1.05)	0.595			0.93 (0.88–0.98)	0.011
Moderate instability			1.09 (1.01–1.18)	0.029			0.89 (0.83–0.96)	0.003
High to very high instability			1.05 (0.93–1.18)	0.442			0.82 (0.73–0.92)	0.001

CHESS values to range from 0 (no health instability) to 4 (high to very high instability), with higher values indicative of adverse outcomes.

CC, chronic condition; CHESS, Changes in Health, End-stage disease and Symptoms and Signs; ED, emergency department; MDS-HSI, Minimum Data Set Health Status Index; sHR, sub-hazard ratio.

Of note in the multivariable models, men (versus women) had a greater risk of hospitalization (sHR = 1.18, 95% CI: 1.13–1.24) but lower risk of an ED visit that did not result in an inpatient stay (sHR = 0.94, 95% CI: 0.90–0.99). For both outcomes, sub-hazards were not linearly associated with income quintile. Hospitalization risk was no different by rurality, but risk of an ED visit was greater among rural (versus urban) residents (sHR = 1.18, 95% CI: 1.12–1.25). Additionally, clients with previous hospitalizations in the past year were significantly more likely to be admitted to hospital during follow-up but not more likely to experience an ED visit (e.g., for clients with 2+ recent hospitalizations compared with none, sHRs were 1.40, 95% CI: 1.31–1.49, and 1.04, 95% CI: 0.98–1.11, for hospitalization and ED visit risk, respectively, from multivariable analyses). Previous ED visits were associated with a significantly greater risk for both outcomes (e.g., for clients with 2+ recent ED visits compared to none, sHRs were 1.39, 95% CI: 1.32–1.47, and 1.94, 95% CI: 1.85–2.04, for hospitalization and ED visit risk, respectively).

**Fig 1:**
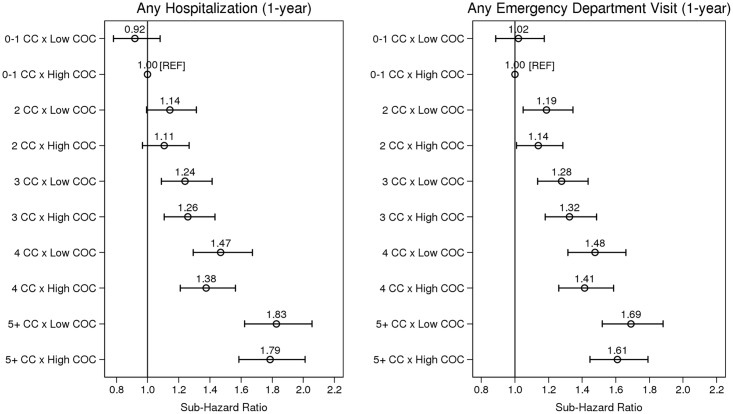
The association between level of multimorbidity and 1-y risk of acute hospitalization and emergency department visit as modified by continuity of care. Sub-hazard ratios account for the competing risks of death and long-term care admission. Estimates adjusted for age, sex, income, marital status, rurality, prior hospitalizations and ED visits, Minimum Data Set Health Status Index, and Changes in Health, End-stage disease and Symptoms and Signs scale. CC, chronic condition; COC, continuity of physician care.

Adjusted for age and sex only, low (versus high) COC was associated with an 11% increased risk of hospitalization (sHR = 1.11, 95% CI: 1.07–1.16) and a 7% increased risk for an ED visit (sHR = 1.07, 95% CI: 1.03–1.11). These associations were not statistically significant, however, in the full multivariable models (Table 3). Fig 1 illustrates the effect modification by COC on level of multimorbidity for both outcomes, Table 4 presents sHRs for low (versus high) COC at each level of multimorbidity (reference = 0–1 chronic conditions), and S1 Fig. shows cumulative incidence estimates. Although point estimates diverged, no comparisons were statistically significant; in other words, COC did not statistically (using a *p <* 0.05 criterion) modify the association between level of multimorbidity and either outcome. Wald tests for significance of the interaction terms confirmed this finding (*p =* 0.566 for hospitalizations and *p =* 0.637 for ED visits).

**Table 4:** Sub-hazard ratios and 95% confidence intervals representing the effect modification of low (versus high, reference) continuity of care at each level of multimorbidity (from multivariable regression).

Level of multimorbidity	Outcome: hospitalization		Outcome: ED visit	
	sHR (95% CI) for low COC (ref = high)	*p*-Value	sHR (95% CI) for low COC (ref = high)	*p*-Value
0–1 CCs	0.92 (0.76–1.01)	0.299	1.02 (0.89–1.18)	0.779
2 CCs	1.13 (0.92–1.38)	0.251	1.02 (0.86–1.22)	0.819
3 CCs	1.07 (0.89–1.30)	0.464	0.94 (0.80–1.12)	0.508
4 CCs	1.16 (0.96–1.41)	0.116	1.02 (0.87–1.21)	0.784
5 CCs	1.11 (0.94–1.33)	0.223	1.03 (0.88–1.20)	0.712

Models adjust for all covariates: age, sex, income, marital status, rurality, prior hospitalizations, prior emergency department use, Minimum Data Set Health Status Index, Changes in Health, End-stage disease and Symptoms and Signs scale, plus interaction between COC and level of multimorbitiy used to derive associations.

CC, chronic condition; COC, continuity of physician care; sHR, sub-hazard ratio.

The results from multiple sensitivity analyses are presented in S6 Table and S2 and S3 Figs. In each model tested, there were no differences in data interpretation to that of our primary analyses.

## Discussion

In our investigation of older community residents with dementia receiving home care services in Ontario, Canada, we observed a large burden of comorbid chronic disease in addition to dementia, and this multimorbidity was associated with an increased risk of subsequent hospital admission and ED visit. In multivariable analyses that accounted for competing risks of death and LTC admission, risks of all-cause hospitalization and ED visit both increased monotonically with each additional diagnosis, and were 88% greater and 63% greater, respectively, among those with five or more comorbid conditions relative to those with dementia alone or with dementia and one other condition. In this population, low physician continuity was associated with elevated risk of hospitalization and ED use in age- and sex-adjusted analyses, but not in multivariate models. Contrary to our hypothesis, greater COC did not modify the association between level of multimorbidity and hospitalization or ED visit risk in this older population with dementia.

In this study, more than one-quarter of otherwise eligible long-stay home care clients aged 50 y and older were diagnosed with dementia. This prevalence is comparable to that found in other studies of the home care sector in Ontario [21,22], and larger than in the general population in the province [36] or elsewhere [37]. The high prevalence of multimorbidity we observed in our cohort is consistent with previous studies on comorbidity among persons with dementia residing in a community setting [2,38,39], where hypertension, osteoarthritis, and diabetes are commonly reported. More than one-third of our study population had five or more chronic conditions in addition to dementia, and only 11% of our sample had dementia alone or with one other condition. Multimorbidity, therefore, is very much the norm rather than the exception in this group of patients.

Detailed examinations of the relationships between multimorbidity with health service utilization within the dementia population are currently limited. Bynum et al. [9] described crude rates of hospitalization for all causes and for ambulatory-care-sensitive conditions stratified by number of chronic conditions. As in the current work, outcomes were more frequent with each additional condition. These findings have important implications for patients with dementia, their families and care providers, and healthcare policymakers. They highlight the need to develop effective interventions targeted to the particular needs of this population. Unique challenges exist in detecting and managing comorbidities for dementia patients [40], including difficulties in communicating medical complaints because of memory and language deficits. Clinical encounters may also be dominated by dealing with dementia and its manifestations, to the exclusion of other conditions. While patient-centered care embracing the principles of chronic disease management should be encouraged [41], the nature of dementia will in itself make this more difficult.

A component of chronic disease management and improved health system integration, measures of COC quantify the dispersion of physician visits among providers. We found that physician continuity decreased with increasing multimorbidity in the dementia population. Interestingly, low (versus high) COC was associated with an 11% increase in hospitalization risk and a 7% increase in ED visit risk in age- and sex-adjusted analyses, but results were nonsignificant in the fully adjusted models. We also found that COC did not modify the associations between level of multimorbidity and hospitalization and ED visit outcomes, a finding verified in multiple sensitivity analyses. In contrast, COC was found to modify observed associations between multimorbidity and hospitalization (adjusting for age and sex) in the general population of Ontario [23], and low levels of COC have been associated with higher rates of hospitalization, ED visits, imaging and laboratory testing, and healthcare spending among dementia patients (relative to propensity-matched Medicare beneficiaries) in the Unites States [20]. Although comparisons between studies are limited due to differences in study populations, together these findings indicate that the relationship between multimorbidity, COC, and healthcare is complex. For dementia care in particular, careful attention will have to be placed on the quality, appropriateness, and quantity of interactions between the lead practitioner, the patient, family caregivers, and other components of the healthcare system such as home care delivery.

In this regard, there are multiple targets for future research. Detailed examination of the longitudinal relationships between the intensity of (formal and informal) long-stay home care services and physician continuity may provide insights into our null findings. Both informational and management continuity [42] may play a role as effect modifiers of the relationship between multimorbidity and health service use, but this has yet to be explored. In addition, differences in health system access may exist in rural versus urban areas, which has implications for home care use, frequency of physician visits, and use of acute and emergency services. We were unable to explore these differences in the current work as most (88%) of our study cohort resided in urban areas. Exploring these and other areas could be useful in identifying which individuals with dementia are most at risk and in need of intervention.

This study has several limitations requiring comment. This study was a retrospective cohort analysis of routinely collected health information and is therefore subject to limitations arising from the nature of the data [43]. We treated multimorbidity level and COC as fixed covariates in all analyses. Inclusion of time-varying variables in competing risk regression, however, can result in biased estimation [44]. We focused only on all-cause hospitalizations, without stratifying by specific reasons for hospitalization and ED visits. Such stratification could potentially inform interventions. We also focused only on clients with dementia. The effects of multimorbidity and physician continuity on a population without dementia may be different. Future studies comparing persons with versus without dementia may help to explain our findings. Strengths of this work include a large and representative population-based sample of community residents with dementia in the home care sector. Results would be expected to be generalizable to other jurisdictions with comparable healthcare systems that provide similar community-based services to persons with dementia. Whether our findings also apply to those living with dementia in the community who have not yet presented to the healthcare system is unknown and should be investigated in future studies. We used validated algorithms to define many chronic diagnoses, and the conditions we investigated are also consistent with those explored by other researchers and governing bodies to understand multimorbidity elsewhere [45,46]. Our models considered multiple competing risks and also adjusted for multiple but independent disease severity indicators that are not readily available in traditional health administrative databases. Linking to the RAI-HC data enabled this inclusion.

### Conclusions

With increases in life expectancy and improvements to disease detection, the number of individuals living in the community with dementia and multimorbidity will increase. Our findings regarding the distribution of chronic disease burden and associated elevated risks of acute hospital admission and ED visits may be useful for healthcare providers and policymakers in identifying at-risk individuals with dementia in the community and setting priorities for care strategies. Heightened relational physician continuity did not modify the associations in this study. Additional research is therefore warranted to identify modifiable health system and other factors predictive of health outcomes to facilitate the development of effective interventions aimed at reducing costly health system use for this complex population.

## Supporting information

S1 Fig Cumulative incidence estimates of 1-y risk of hospitalization and emergency department visit. (PDF)

S2 Fig The association between level of multimorbidity and 1-y risk of acute hospitalization and emergency department visit as modified by continuity of care, including only individuals with three or more physician visits (sensitivity analysis 3). (PDF)

S3 Fig The association between level of multimorbidity and 1-y risk of acute hospitalization and emergency department visit as modified by continuity of care defined by tertiles (sensitivity analysis 4). (PDF)

S1 Table Description of the health administrative datasets used for this research. (PDF)

S2 Table List of diagnostic information for defining dementia and the 16 selected chronic conditions under investigation in this study. (PDF)

S3 Table Counts per study inclusion criterion. (PDF)

S4 Table Prevalence of 16 chronic conditions by level of multimorbidity. (PDF)

S5 Table Proportion of long-stay home care clients with dementia in Ontario in 2012 who experienced each outcome under investigation during the 1-y follow-up period, by low and high continuity of care. (PDF)

S6 Table Results from sensitivity analyses 1 and 2 (subset of study population). (PDF)

S1 Text RECORD statement (cohort studies). (PDF)

S2 Text Study protocol. (PDF)

## Abbreviations

CHESS: Changes in Health, End-stage disease and Symptoms and Signs
COC: continuity of physician care
DAD: Discharge Abstract Database
ED: emergency department
LTC: long-term care
MDS-HSI: Minimum Data Set Health Status Index
RAI-HC: Resident Assessment Instrument for Home Care
sHR: sub-hazard ratio

## References

[1] Alzheimer’s Association. 2015 Alzheimer’s disease facts and figures. Alzheimers Dement. 2015;11(3):332–84. 25984581 10.1016/j.jalz.2015.02.003

[2] Bauer K, Schwarzkopf L, Graessel E, Holle R. A claims data-based comparison of comorbidity in individuals with and without dementia. BMC Geriatrics. 2014;14(1):10. 24472217 10.1186/1471-2318-14-10 PMC3909381

[3] Savva GM, Stephan BCM, Alzheimer’s Society Vascular Dementia Systematic Review Group. Epidemiological studies of the effect of stroke on incident dementia: a systematic review. Stroke. 2009;41(1):e41–6. 10.1161/STROKEAHA.109.559880 19910553

[4] Whitmer RA, Karter AJ, Yaffe K, Quesenberry CP, Selby JV. Hypoglycemic episodes and risk of dementia in older patients with type 2 diabetes mellitus. JAMA. 2009;301(15):1565–72. 10.1001/jama.2009.460 19366776 PMC2782622

[5] Bunn F, Burn A-M, Goodman C, Rait G, Norton S, Robinson L, et al Comorbidity and dementia: a scoping review of the literature. BMC Med. 2014;12(1):192. 25358236 10.1186/s12916-014-0192-4 PMC4229610

[6] Lyketsos CG. Prevention of unnecessary hospitalization for patients with dementia. JAMA. 2012;307(2):197–8. 10.1001/jama.2011.2005 22235092

[7] Maxwell CJ, Amuah JE, Hogan DB, Cepoiu-Martin M, Gruneir A, Patten SB, et al Elevated hospitalization risk of assisted living residents with dementia in Alberta, Canada. J Am Med Dir Assoc. 2015;16(7):568–77. 10.1016/j.jamda.2015.01.079 25717011

[8] Phelan EA, Borson S, Grothaus L, Balch S, Larson EB. Association of incident dementia with hospitalizations. JAMA. 2012;307(2):165–72. 10.1001/jama.2011.1964 22235087 PMC3312921

[9] Bynum JPW, Rabins PV, Weller W, Niefeld M, Anderson GF, Wu AW. The relationship between a dementia diagnosis, chronic illness, medicare expenditures, and hospital use. J Am Geriatr Soc. 2004;52(2):187–94. 14728626 10.1111/j.1532-5415.2004.52054.x

[10] Zhao Y, Kuo T-C, Weir S, Kramer MS, Ash AS. Healthcare costs and utilization for Medicare beneficiaries with Alzheimer’s. BMC Health Serv Res. 2008;8(1):1119–8. 10.1186/1472-6963-8-108 PMC2424046 18498638

[11] Feng Z, Coots LA, Kaganova Y, Wiener JM. Hospital and ED use among medicare beneficiaries with dementia varies by setting and proximity to death. Health Aff (Millwood). 2014;33(4):683–90. 24711331 10.1377/hlthaff.2013.1179

[12] LaMantia MA, Stump TE, Messina FC, Miller DK, Callahan CM. emergency department use among older adults with dementia. Alzheimer Dis Assoc Disord. 2016;30(1):35–40. 10.1097/WAD.0000000000000118 26523710 PMC4764430

[13] LaMantia MA, Lane KA, Tu W, Carnahan JL, Messina F, Unroe KT. Patterns of emergency department use among long-stay nursing home residents with differing levels of dementia severity. J Am Med Dir Assoc. 2016;17(6):541–6. 10.1016/j.jamda.2016.02.011 27052563 PMC4884504

[14] Phelan EA, Debnam KJ, Anderson LA, Owens SB. A systematic review of intervention studies to prevent hospitalizations of community-dwelling older adults with dementia. Med Care. 2015;53(2):207–13. 10.1097/MLR.0000000000000294 25588136 PMC4310672

[15] Seitz DP, Gill SS, Gruneir A, Austin PC, Anderson GM, Bell CM, et al Effects of dementia on postoperative outcomes of older adults with hip fractures: a population-based study. J Am Med Dir Assoc. 2014;15(5):334–41. 10.1016/j.jamda.2013.12.011 24524851

[16] Tropea J, LoGiudice D, Liew D, Gorelik A, Brand C. Poorer outcomes and greater healthcare costs for hospitalised older people with dementia and delirium: a retrospective cohort study. Int J Geriatr Psychiatry. 2016 4 25. 10.1002/gps.4491 27114271

[17] Callahan CM, Arling G, Tu W, Rosenman MB, Counsell SR, Stump TE, et al Transitions in care for older adults with and without dementia. J Am Geriatr Soc. 2012;60(5):813–20. 10.1111/j.1532-5415.2012.03905.x 22587849 PMC3354737

[18] Sampson EL, Leurent B, Blanchard MR, Jones L, King M. Survival of people with dementia after unplanned acute hospital admission: a prospective cohort study. Int J Geriatr Psychiatry. 2012;28(10):1015–22. 10.1002/gps.3919 23280594

[19] Reppas-Rindlisbacher CE, Fischer HD, Fung K, Gill SS, Seitz D, Tannenbaum C, et al Anticholinergic drug burden in persons with dementia taking a cholinesterase inhibitor: the effect of multiple physicians. J Am Geriatr Soc. 2016;64(3):492–500. 10.1111/jgs.14034 27000323 PMC4819524

[20] Amjad H, Carmichael D, Austin AM, Chang C-H, Bynum JPW. Continuity of care and health care utilization in older adults with dementia in fee-for-service Medicare. JAMA Intern Med. 2016;176(9):1371–8. 10.1001/jamainternmed.2016.3553 27454945 PMC5061498

[21] Vu M, Hogan DB, Patten SB, Jette N, Bronskill SE, Heckman GA, et al A comprehensive profile of the sociodemographic, psychosocial and health characteristics of Ontario home care clients with dementia. Chronic Dis Inj Can. 2014;34(2–3):132–44. 24991776

[22] Mondor L, Maxwell CJ, Bronskill SE, Gruneir A, Wodchis WP. The relative impact of chronic conditions and multimorbidity on health-related quality of life in Ontario long-stay home care clients. Qual Life Res. 2016;25(10):2619–32. 10.1007/s11136-016-1281-y 27052421

[23] Gruneir A, Bronskill SE, Maxwell CJ, Bai YQ, Kone AJ, Thavorn K, et al The association between multimorbidity and hospitalization is modified by individual demographics and physician continuity of care: a retrospective cohort study. BMC Health Serv Res. 2016;16:154 10.1186/s12913-016-1415-5 27122051 PMC4848783

[24] Jaakkimainen RL, Bronskill SE, Tierney MC, Herrmann N, Green D, Young J, et al Identification of physician-diagnosed Alzheimer’s disease and related dementias in population-based administrative data: a validation study using family physicians’ electronic medical records. J Alzheimers Dis. 2016;54(1):337–49. 10.3233/JAD-160105 27567819

[25] Koné Pefoyo AJ, Bronskill SE, Gruneir A, Calzavara A, Thavorn K, Petrosyan Y, et al The increasing burden and complexity of multimorbidity. BMC Public Health. 2015;15(1):415. 25903064 10.1186/s12889-015-1733-2 PMC4415224

[26] Lane NE, Maxwell CJ, Gruneir A, Bronskill SE, Wodchis WP. Absence of a socioeconomic gradient in older adults’ survival with multiple chronic conditions. EBioMedicine. 2015;2(12):2094–100. 10.1016/j.ebiom.2015.11.018 26844290 PMC4703730

[27] Public Health Agency of Canada. The Chief Public Health Officer’s report on the state of public health in Canada, 2010. Ottawa: Public Health Agency of Canada; 2010 [cited 2017 Feb 7]. http://www.phac-aspc.gc.ca/cphorsphc-respcacsp/2010/fr-rc/pdf/cpho_report_2010_e.pdf.

[28] Public Health Agency of Canada. Economic burden of illness in Canada, 2005–2008. Ottawa: Public Health Agency of Canada; 2014 [cited 2017 Feb 7]. http://www.phac-aspc.gc.ca/publicat/ebic-femc/2005-2008/assets/pdf/ebic-femc-2005-2008-eng.pdf.

[29] Canadian Institute for Health Information. Seniors and the health care system: what is the impact of multiple chronic conditions? Ottawa: Public Health Agency of Canada; 2011 [cited 2017 Feb 7]. https://secure.cihi.ca/free_products/air-chronic_disease_aib_en.pdf.

[30] Wodchis WP, Hirdes JP, Feeny DH. Health-related quality of life based on the minimum data set. Int J Technol Access Health Care. 2003;19(3):490–506. 10.1017/s0266462303000424 12962335

[31] Hirdes JP, Frijters DH, Teare GF. The MDS-CHESS scale: a new measure to predict mortality in institutionalized older people. J Am Geriatr Soc. 51(1):96–100. 12534853 10.1034/j.1601-5215.2002.51017.x

[32] Campitelli MA, Bronskill SE, Hogan DB, Diong C, Amuah JE, Gill S, et al The prevalence and health consequences of frailty in a population-based older home care cohort: a comparison of different measures. BMC Geriatrics. 2016;16(1):133. 27388294 10.1186/s12877-016-0309-z PMC4937594

[33] Bice TW, Boxerman SB. A quantitative measure of continuity of care. Med Care. 1977;15(4):347–9. 859364 10.1097/00005650-197704000-00010

[34] Kralj B. Measuring rurality—RIO2008 BASIC: methodology and results. Toronto: Ontario Medical Association; 2009.

[35] Fine JP, Gray RJ. A proportional hazards model for the subdistribution of a competing risk. J Am Stat Assoc. 1999;94(446):496–509.

[36] Ng R, Maxwell CJ, Yates EA, Nylen K, Antflick J, Jette N, et al. Brain disorders in Ontario: prevalence, incidence and costs from health administrative data. Toronto: Institute for Clinical Evaluative Sciences; 2015 [cited 2017 Feb 7]. http://www.ices.on.ca/~/media/Files/Atlases-Reports/2015/Brain-Disorders-in-Ontario/Full-Report.ashx.

[37] Prince M, Bryce R, Albanese E, Wimo A, Ribeiro W, Ferri CP. The global prevalence of dementia: a systematic review and metaanalysis. Alzheimers Dement. 2013;9(1):63–75.e2. 10.1016/j.jalz.2012.11.007 23305823

[38] Schubert CC, Boustani M, Callahan CM, Perkins AJ, Carney CP, Fox C, et al Comorbidity profile of dementia patients in primary care: are they sicker? J Am Geriatr Soc. 2005;54(1):104–9. 10.1111/j.1532-5415.2005.00543.x 16420205

[39] Poblador-Plou B, Calderón-Larrañaga A, Marta-Moreno J, Hancco-Saavedra J, Sicras-Mainar A, Soljak M, et al Comorbidity of dementia: a cross-sectional study of primary care older patients. BMC Psychiatry. 2014;14(1):84. 24645776 10.1186/1471-244X-14-84 PMC3994526

[40] Fox C, Smith T, Maidment I, Hebding J, Madzima T, Cheater F, et al The importance of detecting and managing comorbidities in people with dementia? Age Ageing. 2014;43(6):741–3. 10.1093/ageing/afu101 25038831

[41] Callahan CM, Schubert CC. Dementia: the complexities of comorbidity in dementia. Nat Rev Neurol. 2014;10(4):184–6. 10.1038/nrneurol.2014.46 24638134

[42] Haggerty JL, Reid RJ, Freeman GK, Starfield BH, Adair CE, McKendry R. Continuity of care: a multidisciplinary review. BMJ. 2003;327(7425):1219–21. 10.1136/bmj.327.7425.1219 14630762 PMC274066

[43] Benchimol EI, Smeeth L, Guttmann A, Harron K, Moher D, Petersen I, et al The REporting of studies Conducted using Observational Routinely-collected health Data (RECORD) statement. PLoS Med. 2015;12(10):e1001885 10.1371/journal.pmed.1001885 26440803 PMC4595218

[44] Latouche A, Porcher R, Chevret S. A note on including time-dependent covariate in regression model for competing risks data. Biom J. 2005;47(6):807–14. 16450853 10.1002/bimj.200410152

[45] Lochner K, Goodman R, Posner S, Parekh A. Multiple chronic conditions among Medicare beneficiaries: state-level variations in prevalence, utilization, and cost, 2011. Medicare Medicaid Res Rev. 2013;3(3). 10.5600/mmrr.003.03.b02 PMC3983735 24753976

[46] Goodman RA, Posner SF, Huang ES, Parekh AK, Koh HK. Defining and measuring chronic conditions: imperatives for research, policy, program, and practice. Prev Chronic Dis. 2013;10:E66 10.5888/pcd10.120239 23618546 PMC3652713

