# Supplementary material for: Multimorbidity and healthcare utilization among home care clients with dementia in Ontario, Canada: A retrospective analysis of a population-based cohort
Source: PLoS Med. 2017 Mar 7;14(3):e1002249. doi: 10.1371/journal.pmed.1002249 (PMC5340355; doi:10.1371/journal.pmed.1002249)

S1 Fig. Cumulative incidence estimates of 1-y risk of hospitalization and emergency department visit.

Data derived from multivariable competing-risks regression (including interaction between continuity of care (COC) and level of multimorbidity)

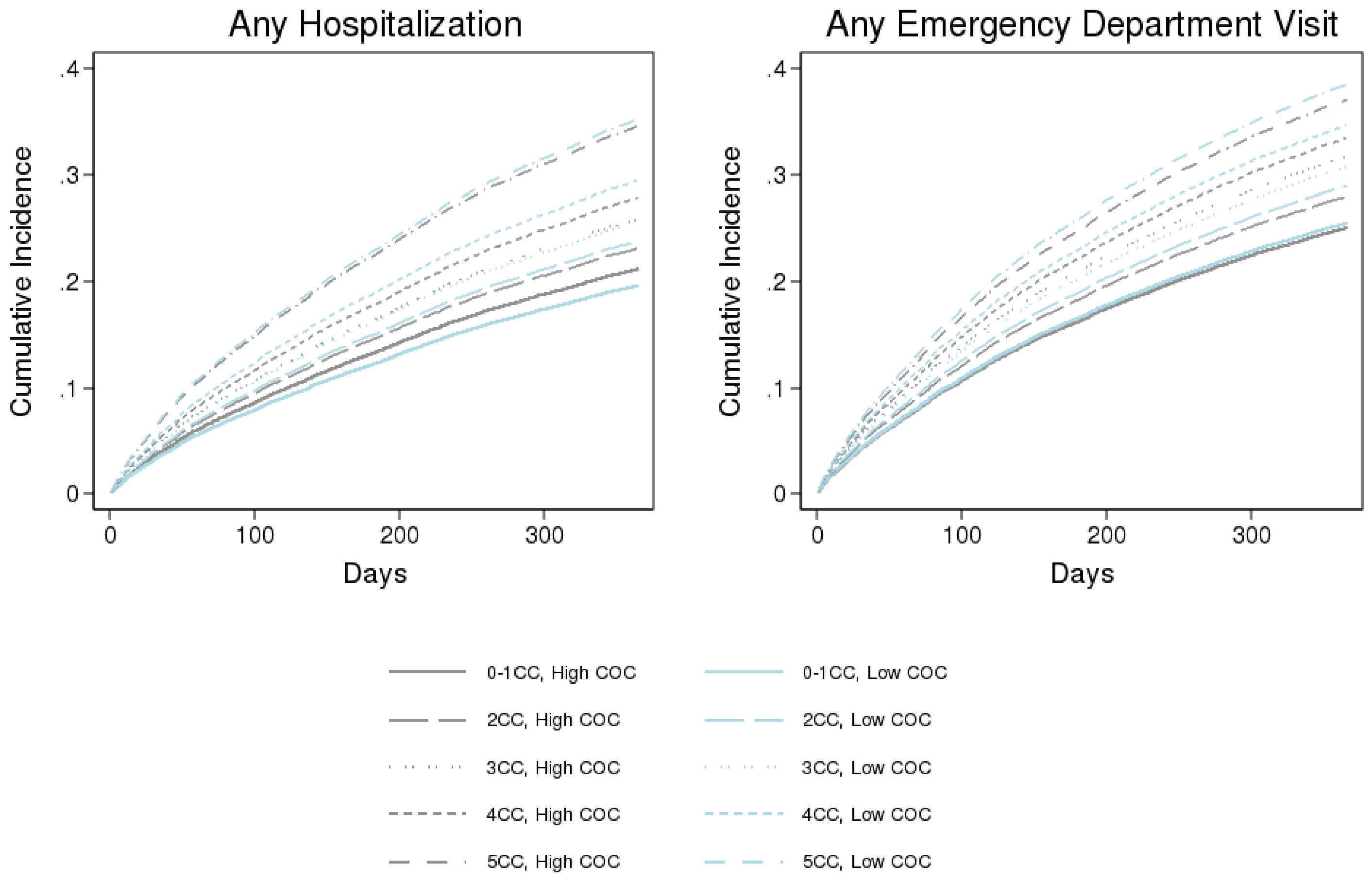

Supplement: S1 Fig — (PDF) [file pmed.1002249.s001.pdf]
