# Supplementary material for: Multimorbidity and healthcare utilization among home care clients with dementia in Ontario, Canada: A retrospective analysis of a population-based cohort
Source: PLoS Med. 2017 Mar 7;14(3):e1002249. doi: 10.1371/journal.pmed.1002249 (PMC5340355; doi:10.1371/journal.pmed.1002249)
Supplement: S2 Fig — (PDF) [file pmed.1002249.s002.pdf]

S2 Fig. The association between level of multimorbidity and 1-y risk of acute hospitalization and emergency department visit as modified by continuity of care, including only individuals with three or more physician visits (sensitivity analysis 3).

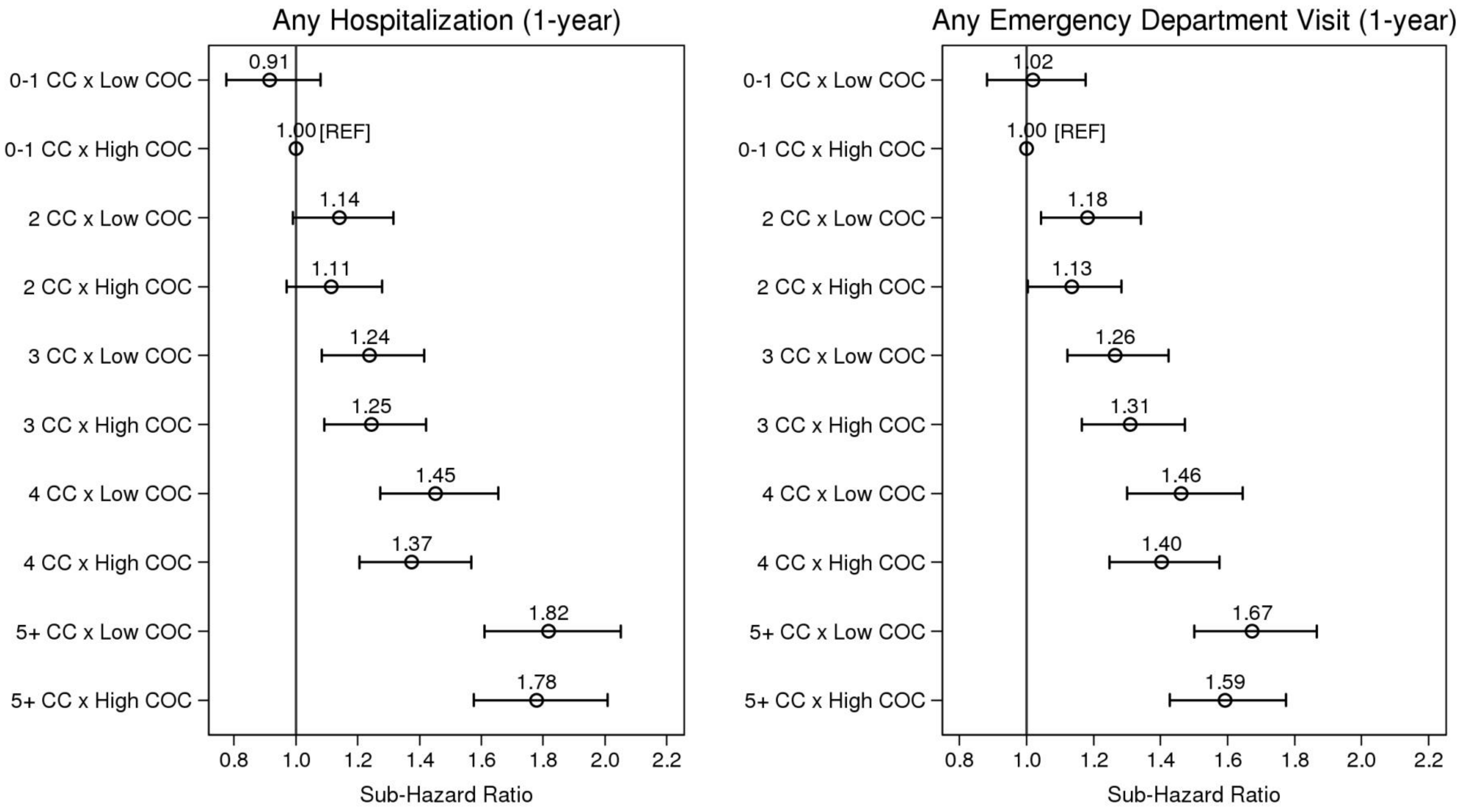

Notes:
CC = Chronic Conditions; COC = Continuity of Care
Sub-Hazard Ratios account for the competing risks of death and LTC admission
Estimates adjusted for age, sex, income, marital status, rurality, prior hospitalizations and ED visits, MDS-HSI and CHESS score
Study population restricted to individuals with 3 or more physician visits (median COC=0.63)
