# Supplementary material for: Multimorbidity and healthcare utilization among home care clients with dementia in Ontario, Canada: A retrospective analysis of a population-based cohort
Source: PLoS Med. 2017 Mar 7;14(3):e1002249. doi: 10.1371/journal.pmed.1002249 (PMC5340355; doi:10.1371/journal.pmed.1002249)
Supplement: S3 Fig — (PDF) [file pmed.1002249.s003.pdf]

S3 Fig. The association between level of multimorbidity and 1-y risk of acute hospitalization and emergency department visit as modified by continuity of care defined by tertiles (sensitivity analysis 4).

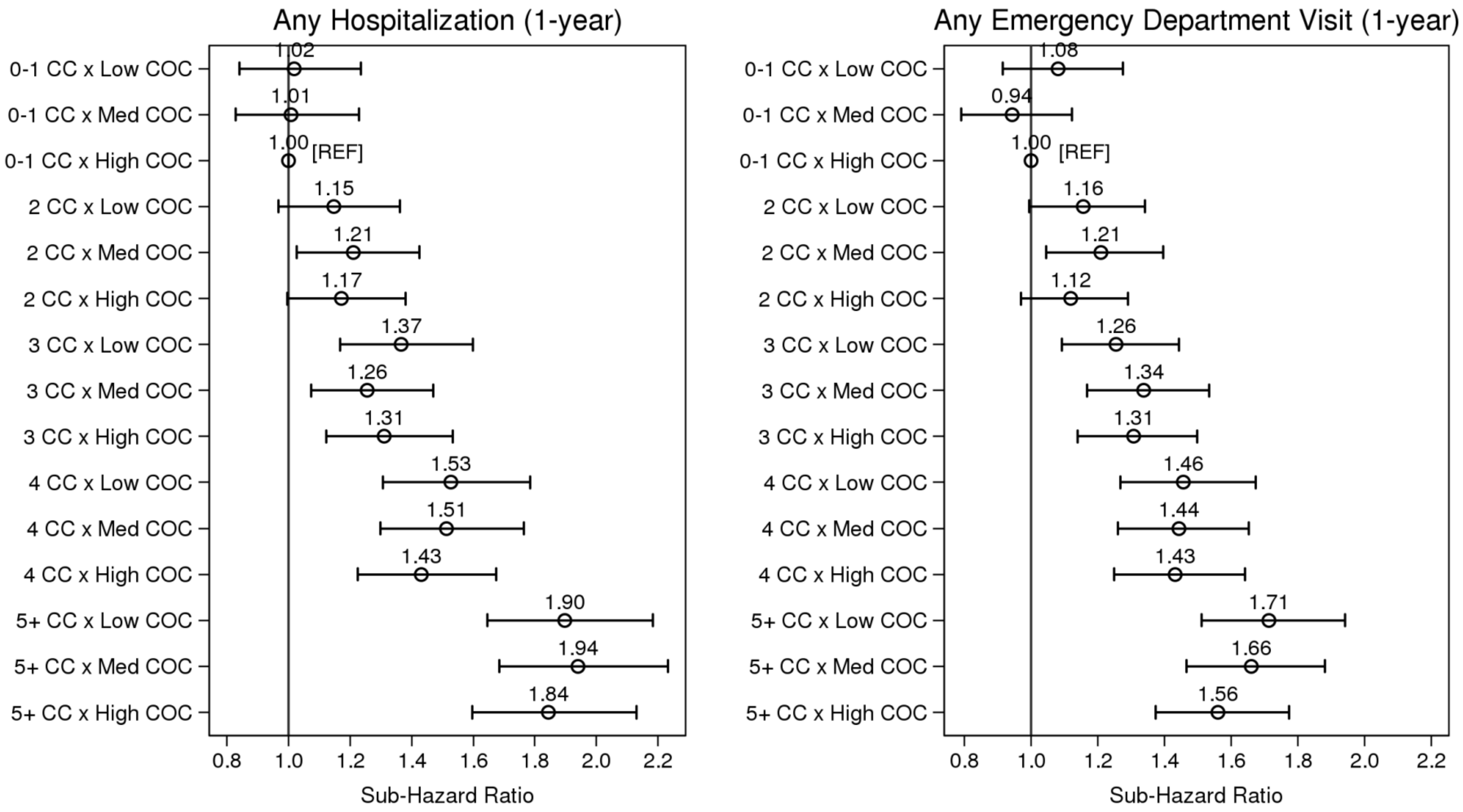

Notes:
CC = Chronic Conditions; COC = Continuity of Care
Sub-Hazard Ratios account for the competing risks of death and LTC admission
Estimates adjusted for age, sex, income, marital status, rurality, prior hospitalizations and ED visits, MDS-HSI and CHESS score
Continuity of care defined by tertiles (Low: 0.00-0.47, Medium: 0.48-0.79, High:0.80-1.00)
