# Supplementary material for: Multimorbidity and healthcare utilization among home care clients with dementia in Ontario, Canada: A retrospective analysis of a population-based cohort
Source: PLoS Med. 2017 Mar 7;14(3):e1002249. doi: 10.1371/journal.pmed.1002249 (PMC5340355; doi:10.1371/journal.pmed.1002249)
Supplement: S1 Table — (PDF) [file pmed.1002249.s004.pdf]

S1 Table. Description of the health administrative datasets used for this research

Datasets used in this research include the Registered Persons Database (RPDB) for patient demographics; the Canadian Institute for Health Information's Discharge Abstract Database (DAD) and National Ambulatory Care Reporting System (NACRS) for identification of inpatient discharges and ED visits, respectively; the Ontario Health Insurance Plan (OHIP) data for physician billings; the Ontario Drug Benefits (ODB) database for prescriptions dispensed from community pharmacies; the Resident Assessment Instrument for Home Care (RAI-HC), a detailed clinical assessment administered by trained case managers at regular intervals to long-stay home care clients (publicly-funded); the Continuing Care Reporting System (CCRS) for LTC placements; and the Statistics Canada 2006 census for area-level data not routinely collected with administrative data sources. These data were linked using unique encoded identifiers and analyzed at the Institute for Clinical Evaluative Sciences (ICES) in Toronto.

Dataset	Description
Registered Persons Database (RPDB)	A population-based registry maintained by the Ontario Ministry of Health and Long-Term Care that contains demographic information (including age, sex, area of residence, dates of birth and where applicable, death) for all individuals who register for health insurance in Ontario
Canadian Institute for Health Information Discharge Abstract Database (DAD)	Contains detailed information abstracted from hospital records from all acute care centers in the province of Ontario. Psychiatric and rehabilitation admissions are contained in other health administrative datasets.
National Ambulatory Care Reporting System (NACRS)	Includes information on hospital- and community-based ambulatory care services provided in the province of Ontario, used specifically in this study to obtain information on patient visits to emergency departments
Ontario Health Insurance Plan (OHIP)	Contains all claims made by all physicians for services provided to Ontario residents
Ontario Drug Benefits (ODB) Database	Contains all claims for prescription drugs dispensed from community pharmacies that are covered under the ODB program. Data is limited to individuals aged ≥65 years or those on social assistance.
Resident Assessment Instrument for Home Care (RAI-HC)	A detailed clinical assessment administered by trained case managers at regular intervals (recommended every 6 months) to all long-stay home care (publicly funded) clients in Ontario. Data include clinical, functional and resource utilization information and are used to inform client needs. The assessment is mandated for all long stay home care clients in Ontario, and each assessment is maintained in the RAI-HC database.
Continuing Care Reporting System (CCRS)	A detailed clinical assessment containing clinical, functional and resource utilization (placement) information on individuals receiving continuing care services, including long-term care, in Ontario
2006 Canadian Census	Includes area-level markers not captured with traditional health administrative data sources (for example, area-level income)
