# Supplementary material for: Multimorbidity and healthcare utilization among home care clients with dementia in Ontario, Canada: A retrospective analysis of a population-based cohort
Source: PLoS Med. 2017 Mar 7;14(3):e1002249. doi: 10.1371/journal.pmed.1002249 (PMC5340355; doi:10.1371/journal.pmed.1002249)
Supplement: S2 Table — (PDF) [file pmed.1002249.s005.pdf]

S2 Table. List of diagnostic information for defining dementia and the 16 selected chronic conditions under investigation in this study.

These conditions represent a subset of all possible chronic conditions that may be experienced by individuals over a lifetime but represent the most substantial conditions from a population perspective.

**Condition**	**ICD 9 / OHIP**	**ICD 10**	**ODB***
Acute Myocardial Infarction (AMI)	410	I21, I22	
Osteo- and other Arthritis:			
(A) Osteoarthritis	715	M15-M19	
(B) Other Arthritis (includes Synovitis, Fibrositis, Connective tissue disorders, Ankylosing spondylitis, Gout Traumatic arthritis, pyogenic arthritis, Joint derangement, Dupuytren's contracture, Other MSK disorders)	727, 729, 710, 720, 274, 716, 711, 718, 728, 739	M00-M03, M07, M10, M11-M14, M20-M25, M30-M36, M65-M79	
Arthritis - Rheumatoid arthritis	714	M05-M06	
Asthma	493	J45	
(all) Cancers	140-239	C00-C26, C30-C44, C45-C97	
Cardiac Arrhythmia	427 (OHIP) / 427.3 (DAD)	I48.0, I48.1	
Congestive Heart Failure	428	I500, I501, I509	
Chronic Obstructive Pulmonary Disease	491, 492, 496	J41, J43, J44	
Coronary syndrome (excluding AMI)	411-414	I20, I22-I25	
Dementia	290, 331 (OHIP) / 046.1, 290.0, 290.1, 290.2, 290.3, 290.4, 294, 331.0, 331.1, 331.5, F331.82 (DAD)	F00, F01, F02, F03, G30	Cholinesterase Inhibitors
Diabetes	250	E08 - E13	
Hypertension	401, 402, 403, 404, 405	I10, I11, I12, I13, I15	

(Other) Mental Illnesses	291, 292, 295, 297, 298, 299, 301, 302, 303, 304, 305, 306, 307, 313, 314, 315, 319	F04, F050, F058, F059, F060, F061, F062, F063, F064, F07, F08, F10, F11, F12, F13, F14, F15, F16, F17, F18, F19, F20, F21, F22, F23, F24, F25, F26, F27, F28, F29, F340, F35, F36, F37, F430, F439, F453, F454, F458, F46, F47, F49, F50, F51, F52, F531, F538, F539, F54, F55, F56, F57, F58, F59, F60, F61, F62, F63, F64, F65, F66, F67, F681, F688, F69, F70, F71, F72, F73, F74, F75, F76, F77, F78, F79, F80, F81, F82, F83, F84, F85, F86, F87, F88, F89, F90, F91, F92, F931, F932, F933, F938, F939, F94, F95, F96, F97, F98
Mood, anxiety, depression and other nonpsychotic disorders	296, 300, 309, 311	F30, F31, F32, F33, F34 (excl. F34.0), F38, F39, F40, F41, F42, F43.1, F43.2, F43.8, F44, F45.0, F45.1, F45.2, F48, F53.0, F68.0, F93.0, F99
Osteoporosis	733	M81, M82
Renal failure	403, 404, 584, 585, 586, v451	N17, N18, N19, T82.4, Z49.2, Z99.2
Stroke (excluding transient ischemic attack)	430, 431, 432, 434, 436	I60-I64

NOTES:

Abbreviations: ICD = International Classification of Disease; ODB = Ontario Drug Benefit program database; OHIP = Ontario Health Insurance Plan, physician billings database;

All case definitions look back to 2001 to ascertain disease status, with the exception of AMI (1 year prior to RAI-HC assessment), Cancer (2 years), Mood Disorder (2 years) and Other Mental Illnesses (2 years)

AMI, Asthma, COPD, CHF, Dementia, Diabetes and Hypertension are based on validated case algorithms (see Sources 1-7 below, respectively). All other conditions required at least one diagnosis recorded in acute care (CIHI) or two diagnoses recorded in physician billings within a two-year period. Dementia, however, required at least one acute record, three or more physician billings within 2 years and separated by 30 days or more, or any prescription dispensing of a cholinesterase inhibitor (see Reference 24 of manuscript)

*ODB prescription drug records are not available for the majority of persons under the age of 65

Sources:

1. Austin PC, Daly PA, Tu JV. A multicenter study of the coding accuracy of hospital discharge administrative data for patients admitted to cardiac care units in Ontario. American Heart Journal 2002;144:290–6.
2. Gershon AS, Wang C, Guan J, Vasilevska-Ristovska J, Cicutto L, To T. Identifying patients with physician-diagnosed asthma in health administrative databases. Can Respir J 2009;16:183–8.
3. Gershon AS, Wang C, Guan J, Vasilevska-Ristovska J, Cicutto L, To T. Identifying Individuals with Physician Diagnosed COPD in Health Administrative Databases. Copd 2009;6:388–94.
4. Schultz SE, Rothwell DM, Chen Z, Tu K. Identifying cases of congestive heart failure from administrative data: a validation study using primary care patient records. Chronic Diseases and Injuries in Canada 2013;33:160–6.
5. Jaakkimainen RL, Bronskill SE, Tierney MC, Herrmann N, Green D, Young J, et al. Identification of Physician-Diagnosed Alzheimer’s Disease and Related Dementias in Population-Based Administrative Data: A Validation Study Using Family Physicians’ Electronic Medical Records. J Alzheimers Dis. IOS Press; 2016 Aug 10;54(1):337–49
6. Hux JE, Ivis F, Flintoft V, Bica A. Diabetes in Ontario: Determination of prevalence and incidence using a validated administrative data algorithm. Diabetes Care 2002;25:512–6.
7. Tu K, Campbell NR, Chen Z-L, Cauch-Dudek KJ, McAlister FA. Accuracy of administrative databases in identifying patients with hypertension. Open Med 2007;1:e18–26.
