# Supplementary material for: Multimorbidity and healthcare utilization among home care clients with dementia in Ontario, Canada: A retrospective analysis of a population-based cohort
Source: PLoS Med. 2017 Mar 7;14(3):e1002249. doi: 10.1371/journal.pmed.1002249 (PMC5340355; doi:10.1371/journal.pmed.1002249)
Supplement: S3 Table — (PDF) [file pmed.1002249.s006.pdf]

S3 Table. Counts per study inclusion criterion

Step	Inclusion criteria	Records Excluded (n)	Cohort Remaining (n)
1	RAI-HC assessments between January and June 2012	n/a	131,845
2	Valid OHIP card number and eligible for (OHIP) coverage	51	131,794
3	Between 50 and 105 years of age	5,874	125,920
4	Records with data quality issues	124	125,796
5	Resident of Ontario	37	125,759
6	One assessment per home care client (closest to Apr 1, 2012)	16,326	109,433
7	Diagnosed with dementia at time of RAI-HC assessment	79,321	30,112
