# Supplementary material for: Multimorbidity and healthcare utilization among home care clients with dementia in Ontario, Canada: A retrospective analysis of a population-based cohort
Source: PLoS Med. 2017 Mar 7;14(3):e1002249. doi: 10.1371/journal.pmed.1002249 (PMC5340355; doi:10.1371/journal.pmed.1002249)
Supplement: S4 Table — (PDF) [file pmed.1002249.s007.pdf]

S4 Table. Prevalence of 16 chronic conditions by level of multimorbidity

Variable	Overall N=30,112	0-1 CC N=3,309	2 CC N=4,799	3 CC N=5,847	4 CC N=5,589	5+ CC N=10,568
Prevalent Chronic Conditions						
Hypertension	24,812 (82.4%)	1,166 (35.2%)	3,467 (72.2%)	4,944 (84.6%)	5,093 (91.1%)	10,142 (96.0%)
Osteo-arthritis	17,977 (59.7%)	468 (14.1%)	2,026 (42.2%)	3,325 (56.9%)	3,808 (68.1%)	8,350 (79.0%)
Diabetes Mellitus	10,361 (34.4%)	163 (4.9%)	824 (17.2%)	1,718 (29.4%)	2,065 (36.9%)	5,591 (52.9%)
Chronic Coronary Syndrome	9,955 (33.1%)	68 (2.1%)	448 (9.3%)	1,200 (20.5%)	1,889 (33.8%)	6,350 (60.1%)
Mood/ Anxiety Disorders	7,379 (24.5%)	177 (5.3%)	622 (13.0%)	1,141 (19.5%)	1,469 (26.3%)	3,970 (37.6%)
Congestive Heart Failure	6,779 (22.5%)	17 (0.5%)	148 (3.1%)	507 (8.7%)	1,010 (18.1%)	5,097 (48.2%)
Cardiac Arrhythmia	6,484 (21.5%)	38 (1.1%)	217 (4.5%)	648 (11.1%)	1,098 (19.6%)	4,483 (42.4%)
(any) Cancer	5,448 (18.1%)	119 (3.6%)	454 (9.5%)	813 (13.9%)	1,090 (19.5%)	2,972 (28.1%)
COPD	4,971 (16.5%)	56 (1.7%)	206 (4.3%)	455 (7.8%)	743 (13.3%)	3,511 (33.2%)
Stroke	4,598 (15.3%)	34 (1.0%)	258 (5.4%)	564 (9.6%)	908 (16.2%)	2,834 (26.8%)
Asthma	4,428 (14.7%)	38 (1.1%)	184 (3.8%)	445 (7.6%)	786 (14.1%)	2,975 (28.2%)
Renal Failure	4,263 (14.2%)	23 (0.7%)	112 (2.3%)	421 (7.2%)	703 (12.6%)	3,004 (28.4%)
Osteoporosis	4,175 (13.9%)	117 (3.5%)	404 (8.4%)	781 (13.4%)	856 (15.3%)	2,017 (19.1%)
(other) Mental Health	3,084 (10.2%)	51 (1.5%)	179 (3.7%)	401 (6.9%)	548 (9.8%)	1,905 (18.0%)
Rheumatoid Arthritis	1,317 (4.4%)	15 (0.5%)	44 (0.9%)	150 (2.6%)	260 (4.7%)	848 (8.0%)
Acute Myocardial Infarction	352 (1.2%)	suppr.	suppr.	28 (0.5%)	30 (0.5%)	285 (2.7%)

Notes:
CC = Chronic Conditions, suppr. = Cell suppressed due to small size (<5);
