# Supplementary material for: Multimorbidity and healthcare utilization among home care clients with dementia in Ontario, Canada: A retrospective analysis of a population-based cohort
Source: PLoS Med. 2017 Mar 7;14(3):e1002249. doi: 10.1371/journal.pmed.1002249 (PMC5340355; doi:10.1371/journal.pmed.1002249)
Supplement: S5 Table — (PDF) [file pmed.1002249.s008.pdf]

S5 Table. Proportion of long-stay home care clients with dementia in Ontario in 2012 who experienced each outcome under investigation during the 1-y follow-up period, by low and high continuity of care.

**(A) Low COC**		**OVERALL**	**0-1 CC**	**2 CC**	**3 CC**	**4 CC**	**5+ CC**
		N=14,754	N=1,420	N=2,154	N=2,739	N=2,714	N=5,727
Acute Hospitalization							
	Censored	5,252 (35.6%)	578 (40.7%)	833 (38.7%)	1,053 (38.4%)	953 (35.1%)	1,835 (32.0%)
	Event	4,491 (30.4%)	263 (18.5%)	502 (23.3%)	706 (25.8%)	828 (30.5%)	2,192 (38.3%)
	Died	580 (3.9%)	62 (4.4%)	67 (3.1%)	105 (3.8%)	99 (3.6%)	247 (4.3%)
	Admitted to LTC	4,431 (30.0%)	517 (36.4%)	752 (34.9%)	875 (31.9%)	834 (30.7%)	1,453 (25.4%)
Emergency Department Visit							
	Censored	4,153 (28.1%)	467 (32.9%)	671 (31.2%)	835 (30.5%)	769 (28.3%)	1,411 (24.6%)
	Event	5,140 (34.8%)	364 (25.6%)	629 (29.2%)	871 (31.8%)	966 (35.6%)	2,310 (40.3%)
	Died	1,054 (7.1%)	87 (6.1%)	117 (5.4%)	180 (6.6%)	178 (6.6%)	492 (8.6%)
	Admitted to LTC	4,407 (29.9%)	502 (35.4%)	737 (34.2%)	853 (31.1%)	801 (29.5%)	1,514 (26.4%)
Any Competing Risk[1]							
	Died	2,682 (18.2%)	180 (12.7%)	315 (14.6%)	436 (15.9%)	479 (17.6%)	1,272 (22.2%)
	Admitted to LTC	5,541 (37.6%)	610 (43.0%)	902 (41.9%)	1,043 (38.1%)	1,029 (37.9%)	1,957 (34.2%)

Notes:

CC = Chronic Conditions, LTC = Long-term care

[1] Frequencies over the 1-year follow-up period, regardless of first event

(B) High COC	OVERALL	0-1 CC	2 CC	3 CC	4 CC	5+ CC
	N=14,825	N=1,703	N=2,536	N=3,010	N=2,805	N=4,771
Acute Hospitalization						
Censored	5,771 (38.9%)	666 (39.1%)	1,048 (41.3%)	1,236 (41.1%)	1,174 (41.9%)	1,647 (34.5%)
Event	4,135 (27.9%)	331 (19.4%)	554 (21.8%)	755 (25.1%)	772 (27.5%)	1,723 (36.1%)
Died	535 (3.6%)	61 (3.6%)	74 (2.9%)	104 (3.5%)	77 (2.7%)	219 (4.6%)
Admitted to LTC	4,384 (29.6%)	645 (37.9%)	860 (33.9%)	915 (30.4%)	782 (27.9%)	1,182 (24.8%)
Emergency Department Visit						
Censored	4,506 (30.4%)	544 (31.9%)	825 (32.5%)	952 (31.6%)	897 (32.0%)	1,288 (27.0%)
Event	4,910 (33.1%)	422 (24.8%)	710 (28.0%)	963 (32.0%)	968 (34.5%)	1,847 (38.7%)
Died	999 (6.7%)	89 (5.2%)	154 (6.1%)	193 (6.4%)	167 (6.0%)	396 (8.3%)
Admitted to LTC	4,410 (29.7%)	648 (38.1%)	847 (33.4%)	902 (30.0%)	773 (27.6%)	1,240 (26.0%)
Any Competing Risk[1]						
Died	2,504 (16.9%)	227 (13.3%)	340 (13.4%)	477 (15.8%)	446 (15.9%)	1,014 (21.3%)
Admitted to LTC	5,599 (37.8%)	767 (45.0%)	1,042 (41.1%)	1,148 (38.1%)	1,023 (36.5%)	1,619 (33.9%)

Notes:

CC = Chronic Conditions, LTC = Long-term care

[1] Frequencies over the 1-year follow-up period, regardless of first event
