# Supplementary material for: Multimorbidity and healthcare utilization among home care clients with dementia in Ontario, Canada: A retrospective analysis of a population-based cohort
Source: PLoS Med. 2017 Mar 7;14(3):e1002249. doi: 10.1371/journal.pmed.1002249 (PMC5340355; doi:10.1371/journal.pmed.1002249)
Supplement: S6 Table — (PDF) [file pmed.1002249.s009.pdf]

S6 Table. Results from sensitivity analyses 1 and 2 (subset of study population).

Association between level of multimorbidity (number of diagnosed chronic conditions) with 1-year hospitalizations and Emergency Department visits among home care clients with dementia in Ontario (2012) in sensitivity analyses:

Sensitivity 1: Restricting home care clients based on their reason for index RAI-HC assessment (N=28,753 or 95% of original cohort), and

Sensitivity 2: Restricting home care clients based on their cognition level (N=25,618 or 85% of original cohort).

	**Outcome: Hospitalizations**				**Outcome: ED Visits**			
	**Sensitivity 1 sHR (95% CI)**		**Sensitivity 2 sHR (95% CI)**		**Sensitivity 1 sHR (95% CI)**		**Sensitivity 2 sHR (95% CI)**	
Level of Multimorbidity								
0-1 CC	1.00 [REF]		1.00 [REF]		1.00 [REF]		1.00 [REF]	
2 CC	1.17 (1.05-1.30)	*	1.17 (1.04-1.31)	*	1.15 (1.05-1.26)	*	1.18 (1.07-1.30)	*
3 CC	1.31 (1.19-1.45)	*	1.30 (1.17-1.45)	*	1.30 (1.19-1.42)	*	1.33 (1.21-1.46)	*
4 CC	1.48 (1.34-1.63)	*	1.45 (1.30-1.62)	*	1.44 (1.33-1.57)	*	1.45 (1.32-1.60)	*
5+ CC	1.88 (1.72-2.06)	*	1.85 (1.67-2.05)	*	1.63 (1.50-1.77)	*	1.66 (1.52-1.82)	*
Sex								
Women	1.00 [REF]		1.00 [REF]		1.00 [REF]		1.00 [REF]	
Men	1.20 (1.14-1.26)	*	1.21 (1.15-1.27)	*	0.95 (0.90-0.99)	*	0.95 (0.91-1.00)	*
Age (continuous)	1.01 (1.00-1.01)	*	1.01 (1.00-1.01)	*	1.00 (1.00-1.00)		1.00 (0.99-1.00)	
Income Quintile								
1 (low)	1.04 (0.97-1.11)		1.04 (0.97-1.11)		1.06 (0.99-1.13)		1.05 (0.99-1.13)	
2	1.02 (0.95-1.09)		1.00 (0.93-1.07)		1.11 (1.04-1.18)	*	1.09 (1.02-1.16)	*
3	0.96 (0.90-1.03)		0.95 (0.88-1.02)		1.05 (0.98-1.12)		1.01 (0.95-1.09)	
4	0.91 (0.85-0.98)	*	0.91 (0.84-0.98)	*	1.04 (0.97-1.11)		1.03 (0.96-1.10)	
5 (high)	1.00 [REF]		1.00 [REF]		1.00 [REF]		1.00 [REF]	
Marital Status								
Married	1.00 [REF]		1.00 [REF]		1.00 [REF]		1.00 [REF]	
Widowed	0.98 (0.93-1.03)		0.97 (0.92-1.03)		0.95 (0.90-0.99)	*	0.95 (0.91-1.01)	
Separated/Divorced	0.99 (0.90-1.10)		1.00 (0.90-1.10)		0.94 (0.86-1.03)		0.93 (0.85-1.01)	

Never Married/Other	0.86 (0.76-0.97)	*	0.89 (0.79-1.00)		0.85 (0.77-0.95)	*	0.89 (0.80-1.00)	
Residence								
Urban	1.00 [REF]		1.00 [REF]		1.00 [REF]		1.00 [REF]	
Rural	1.04 (0.97-1.11)		1.04 (0.97-1.11)		1.20 (1.13-1.27)	*	1.19 (1.12-1.27)	*
Continuity of Care								
Low (<median)	1.01 (0.97-1.06)	*	1.02 (0.97-1.07)		1.03 (0.99-1.07)		1.02 (0.98-1.06)	
High (>=median)	1.00 [REF]		1.00 [REF]		1.00 [REF]		1.00 [REF]	
Prior Hospitalizations								
None	1.00 [REF]		1.00 [REF]		1.00 [REF]		1.00 [REF]	
1	1.16 (1.10-1.23)	*	1.17 (1.10-1.23)	*	0.96 (0.91-1.00)		0.96 (0.91-1.01)	
≥2	1.44 (1.35-1.54)	*	1.41 (1.31-1.50)	*	1.06 (0.99-1.13)		1.05 (0.99-1.12)	
Prior ED visits								
None	1.00 [REF]		1.00 [REF]		1.00 [REF]		1.00 [REF]	
1	1.23 (1.16-1.30)	*	1.21 (1.15-1.29)	*	1.36 (1.29-1.43)	*	1.37 (1.30-1.45)	*
≥2	1.41 (1.34-1.49)	*	1.40 (1.33-1.48)	*	1.97 (1.88-2.07)	*	1.97 (1.87-2.07)	*
MDS-HSI (continuous)	1.16 (1.03-1.30)	*	1.03 (0.91-1.18)		2.19 (1.97-2.43)	*	1.91 (1.70-2.15)	*
CHESS								
No instability	1.00 [REF]		1.00 [REF]		1.00 [REF]		1.00 [REF]	
Minimal instability	1.09 (1.02-1.15)	*	1.08 (1.01-1.14)	*	1.02 (0.97-1.08)		1.02 (0.96-1.07)	
Low instability	1.01 (0.95-1.08)		1.00 (0.94-1.07)		0.94 (0.89-1.00)	*	0.95 (0.89-1.00)	
Moderate instability	1.10 (1.02-1.20)	*	1.10 (1.02-1.20)	*	0.91 (0.84-0.98)	*	0.89 (0.83-0.97)	*
High-very high instability	1.09 (0.96-1.23)		1.04 (0.91-1.19)		0.82 (0.72-0.93)	*	0.80 (0.70-0.91)	*

Notes:

* denotes $p<0.05$

Abbreviations: sHR = sub-hazard ratio; CI = confidence interval; CC = chronic conditions

CHESS values to range from 0 (no health instability) to 4 (high to very high instability), with higher values indicative of adverse outcomes
