# Supplementary material for: Multimorbidity and healthcare utilization among home care clients with dementia in Ontario, Canada: A retrospective analysis of a population-based cohort
Source: PLoS Med. 2017 Mar 7;14(3):e1002249. doi: 10.1371/journal.pmed.1002249 (PMC5340355; doi:10.1371/journal.pmed.1002249)
Supplement: S2 Text — (PDF) [file pmed.1002249.s011.pdf]

S2 Text: Study Protocol

Multimorbidity and healthcare utilization among home care clients with dementia in Ontario, Canada: a retrospective analysis of a population-based cohort

Authors: Luke Mondor; Colleen J. Maxwell; David B. Hogan; Susan E. Bronskill; Andrea Gruneir; Natasha E Lane; Walter P Wodchis

Dataset Creation Plan

(Summary of document submitted to the Research Ethics Board of Sunnybrook)

**Project Initiation<br>This Section must be Completed Prior to Project Dataset(s) Creation**	
**Project Title:**	Health System Performance Research Network (HSPRN): Multiple Chronic Disease Cohort: Dementia sub-project
**Research Program:**	HSPE
**Site:**	ICES Central
**Project Objectives:**	*Insert Project Objectives as listed in the approved ICES Project PIA*
	To examine the associations between multimorbidity measures (# of chronic conditions) and subsequent 1-year health outcomes (including total and ACSC-related hospitalizations) among home care clients with dementia; and, (ii) to examine whether these associations are modified by HRQL, continuity of care (COC), or by caregiver stress.<br><br>Some plausible hypotheses:<br>o An increasing number of chronic conditions will be associated with increased risk for hospitalization;<br>o Clients with dementia with poorer continuity of care, worse HRQL scores and with caregivers experiencing higher levels of distress will be at greater risk for hospitalization;<br>o The risk of total hospitalization associated with the multimorbidity measures will be greater for dementia clients with poorer continuity of care; worse HRQL scores; and, with caregivers experiencing higher levels of distress;<br><br>*Change: Dropped ACSC hospitalizations from objectives. No standard definition of conditions specific to persons with dementia. Among available definitions, availability of ICD-10 codes are missing. Last, the mean age of the derived cohort was 84 years. Therefore, focus placed only on total/ all-cause events.*<br><br>*Change: Focused here on COC only. Unlike HRQL and caregiver stress, COC is a health system factor that is potentially modifiable.*

Project Initiation<br>This Section must be Completed Prior to Project Dataset(s) Creation		
**ICES Project PIA Initial Approval Date:**	*The ICES Employee or agent who is responsible for creating the Project Dataset(s) is responsible for ensuring there is an approved ICES Project PIA and verifying the date of approval prior to creating the Project Dataset(s)*	
	2015 Feb	
**Principal Investigator (PI):**	Walter Wodchis<br>Colleen Maxwell	
**Is the PI an ICES Student/Trainee?**	☐ ICES Student ☐ ICES Fellow ☐ ICES Post-Doctoral Trainee ☐ Visiting Scholar	
**Project Team Member(s) Responsible for Project Dataset Creation and/or Statistical Analysis (list all):**	*The person(s) named (ICES Analyst, Appointed Analyst, Analytic Epidemiologist, PI, and/or Student) are responsible for creating the Project Dataset(s) and/or statistical analysis*	
	Luke Mondor (luke.mondor@ices.on.ca)<br>Full list of team members: Luke Mondor, Colleen Maxwell, Andrea Gruneir, Susan Bronskill, Natasha Lane*, David Hogan*, Walter Wodchis<br>*NDA completed.	
**Designated ICES Research Practice Staff accountable for Project Documentation:**	*The person named (ICES staff) is accountable for ensuring that the approved ICES Project PIA, PIA Amendments, and DCP are saved on the T Drive, ensuring PIA Amendments are submitted as required, ensuring DCP Amendments are documented, and sharing the final DCP with the PI/Responsible ICES Scientist at project completion*	
	Luke Mondor (luke.mondor@ices.on.ca)	
**DCP Creation Date and Author:**	*Date DCP was finalized prior to Project Dataset(s) creation*	*Name of person who created the DCP*
	Date	***Name***
	April 3, 2015	Luke Mondor

Project Amendments and Reconciliation			
**ICES Project PIA Amendment History:**	*Privacy approval date*	*Person who submitted amendment*	*Note that any changes to the list of ICES Data or Project Objectives require a PIA Amendment*
	Date	***Name***	***Amendment***
	Apr 15 2016	LM	Datasets added
**DCP Amendment History:**	*Date DCP amended*	*Person who made the DCP amendment*	*Note that any DCP amendments involving changes to the list of ICES Data or Project Objectives require a PIA Amendment*
	Date	***Name***	***Amendment***
	Fall 2015	LM	Dropped ACSC hospitalizations due to lack of standard definition for target population (with full list of validated ICD9 and 10 codes). Replaced with any (all-cause) hospitalization.
	Feb 2016	LM	Updated definitions of continuity of care
	Feb 2016	LM	Updated analytical plan
	April 2016	LM	Added data holdings, updated analytical plan

Project Cohort	
**Study Design**	☒ Cohort study ☐ Matched cohort study ☐ Case-control study<br>☐ Cross-sectional study ☐ Other (specify):
**Index Event / Inclusion Criteria**	All individuals administered a RAI-HC assessment between January and June 2012. For IKNs with multiple assessments in the accrual window, select the nearest RAI-HC assessment to April 1, 2012 as the index event.
**Estimated Size of Cohort**	Approx. n=30,000
**Exclusions (in order)**	*Step* Description
	1 Invalid IKNs
	2 Age < 50 or Age > 105
	3 Missing sex
	4 RAI assessment date (r1c) > death date (data quality issues)
	5 Not OHIP eligible on assessment date (r1c, use %getelig)

Project Cohort		
	6	Date of last care (DOLC) < '01Apr2007'd (5years)
	7	Resides in non-Ontario postal code (i.e., substr(pstlcode,1,1,) not in K,L,M,N,P)
	8	NOT Diagnosed with dementia

Project Time Frame Definitions

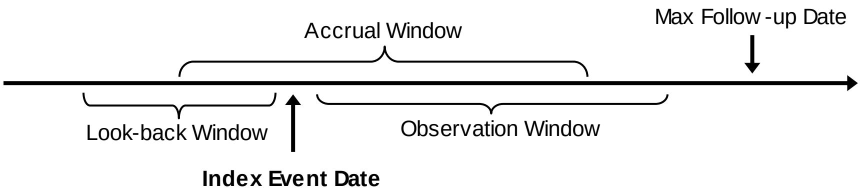

**Accrual Start/End Dates**	January 1, 2012 to June 30, 2012 (i.e., April 1, 2012 ± 90 days)
**Max Follow-up Date**	June 30, 2013
**When does observation window terminate?**	June 30, 2013
**Lookback Window(s)**	To ascertain prevalent disease status (17) diagnoses: April 1, 2003

Variable Definitions	
**Main Exposure or Risk Factor**	Physician-diagnosed chronic conditions (appendix 1)<br>1. Each of 16 conditions<br>2. Number of chronic diagnoses (0-1, 2, 3, 4, 5+ … count excludes the dementia diagnosis)
**Primary Outcome Definition**	Any acute care admission<br>- %getcihi, source=inpatient, acute=T, inclsuspect=T<br>- keep if: R1c <= admdate <= r1c + 365<br>- group by hospital EPISODES<br>- keep first episode, looking at episode admission date<br>- remove any errant observations (ex, if r1c assessment is during a hospital stay)
**Secondary Outcome Definition(s)**	Any ED visit<br>- %getnacrs, source=ed, dedup=T, inclscheduled=F, inclsuspect=T,<br>- keep if: r1c <= regdate <= r1c +365<br>- Exclude those admitted visdisp=6,7; to_type=I<br>- Keep first observation
**Baseline Characteristics**	From Registered Persons Database (use: %getdemo)<br>- Age (years)

Variable Definitions	
	- Sex (M/F)<br>- Neighbourhood-level income quintile (1-5)<br>- Rurality Index of Ontario (Rural v Urban)<br><br>From RAI-HC data<br>- Marital status (categorical, 4)<br>- bb4 = 2 then married<br>- bb4 = 3 then widowed<br>- bb4 = 1,6 then never married/other<br>- bb4 = 4,5 then separated/divorced<br>- bb4 = . then missing<br>- RAI scales **collapse into smaller (meaningful) categories based on distribution<br>- CHESS (instability)<br>- MAPLe (priority) *did not use*<br>- Depression Rating Scale (*a DRS score of 3+ indicates presence of clinically important depressive symptoms) *did not use*<br>- ADL *did not use – included in MDSHSI score*<br>- CPS *did not use – included in MDSHSI score*<br>Caregiver distress (binary) *did not use*<br>- if (g2a = 1 or g2c = 1) then distress = 1<br>- else distress = 0
**Other Variables**	MDS-HSI HRQL utility score (obtained from MDS-HSI)<br>- Single attribute HRQL utility scores (obtained from MDS-HSI macro coding - each variable is categorical, with 4-6 levels<br>- Sensation, Mobility, Emotion, Cognition, Self-Care, Pain<br><br>Continuity of Care: Bice Index (continuous, binary)<br>- Steps:<br>- Obtain all OHIP visits 1-year prior to assessment date (r1c)<br>- Score ranges from 0 (low COC) to 1 (high = saw only same physician over period)<br>- Keep BICE variable and total no. 1-yr physician visits count<br>- Keep var as continuous<br>*REVISED DEFINITION*<br>- *Define second var as binary, based on the median COC score*<br>- *Obtain all OHIP visits 2-year prior to assessment date (r1c). Greater lookback ensures greater stability and reduces the number of observations with missing information.*<br>- *Score ranges from 0 (low COC) to 1 (high = saw only same physician over period)*<br>- *Keep BICE variable and total no. 2-yr physician visits count*<br>- *O/H/L only (ambulatory visits)*

Variable Definitions

- *Keep var as continuous*
- *Define second var as binary, based on the median score (high/low). Modelling interaction of continuous variable presents challenges*
- *Include all individuals that have 1 or more physician visits.*

Death (from %getrpdb)

- Identify all IKNs where r1c < dthdate <= r1c+365 *added*

LTC admission (CCRS-LTC dataset)

- Identify all IKNs where r1c < admdate <= r1c+365 *added*

Health system use, 1yr prior to baseline (where baseline = assessment date)
-no. acute hospital episodes (0,1,2+) – CIHI-DAD
-no. unplanned ed visits (0,1,2+) – NACRS

Analysis Plan and Dummy Tables

Repeat models of: Gruneir, A., Bronskill, S. E., Maxwell, C. J., Bai, Y. Q., Kone, A. J., Thavorn, K., et al. (2016). The association between multimorbidity and hospitalization is modified by individual demographics and physician continuity of care: a retrospective cohort study. BMC Health Services Research, 16(154), 1–9.

Unadjusted and adjusted logistic regression (explanatory) models

1. CCs (and each var) unadjusted, separately
2. CCs (and each var) age-sex adjusted, separately
3. CCs, age, sex, marital status, income, rurality, prior hosps, prior ED vis, COC
4. CCs, age, sex, marital status, income, rurality, prior hosps, prior ED vis, COC*CC
5. CCs, age, sex, marital status, income, rurality, prior hosps, prior ED vis, HRQL
6. CCs, age, sex, marital status, income, rurality, prior hosps, prior ED vis, HRQL*CC
7. CCs, age, sex, marital status, income, rurality, prior hosps, prior ED vis, distress
8. CCs, age, sex, marital status, income, rurality, prior hosps, prior ED vis, distress*CC

In addition, explore: Unadjusted and adjusted Cox models

9. CCs (and each var) unadjusted
10. CCs (and each var) age-sex adjusted
11. CCs, age, sex, marital status, income, rurality, prior hosps, prior ED vis, COC
12. CCs, age, sex, marital status, income, rurality, prior hosps, prior ED vis, COC*CC
13. CCs, age, sex, marital status, income, rurality, prior hosps, prior ED vis, HRQL
14. CCs, age, sex, marital status, income, rurality, prior hosps, prior ED vis, HRQL*CC
15. CCs, age, sex, marital status, income, rurality, prior hosps, prior ED vis, distress
16. CCs, age, sex, marital status, income, rurality, prior hosps, prior ED vis, distress*CC

Plot regression coefficients in Stata using coefplot command (i.e., Forest plot)
Test proportional hazards (Cox)
Test interaction significance: Stata's *lincom* command to determine if diff at 5dx is any different than 0-1dx
Repeat analyses for acute hospitalizations and ED visits

UPDATE: focused only on COC as an effect modifier due to 1) the large volume of information/ hypotheses, and 2) of the three variables, COC is modifiable (health system factor)

UPDATE FEB 2016:
Approximately 18% of the cohort died within 1 year of r1c RAI assessment. Clearly, death is a competing risk in quantifying hospitalizations over the 1 yr follow-up. Both logistic and Cox are insufficient approaches to model the data. Move to Fine-Gray competing risks time to event model.

Outcome, categorical variable:
0 = censored (no event)
1 = event (hosp or ED vis)
2 = died

UPDATE APRIL 2016:
Approx. 38% of the cohort has a LTC admission within 1 year of r1c RAI assessment (regardless of hospitalization and ED visit outcomes). Risk of hospitalization is different among those institutionalized vs. those in the community. LTC admission is a competing risk as well and must be accounted for.

Outcome, categorical variable:
0 = censored (no event)
1 = event (hosp or ED vis)
2 = died
3 = LTC admission

UPDATE APRIL 2016: agreed that multivariable analyses should include covariates for:

1. *Age*
2. *Sex*
3. *Marital status*
4. *Income quintile*
5. *Rurality*
6. *MDS-HSI HRQL score (continuous) – reflects disease severity, includes ADL and CPS scales*
7. *CHESS scale – a second marker of severity, predictive of death, but which includes different components than the MDSHSI*
8. *Prior Hosps*
9. *Prior ED use*
10. *COC *alone then as interaction with CCs*

Analysis Plan and Dummy Tables

UPDATE: Report only age-sex adj. associations, and multivariable results (large volume of output)

Plot Schoenfeld residuals to verify assumptions
Plot cumulative incidence function (CIF)

APRIL 2016 – SENSITIVITY ANALYSES, defined after initial (revised Fine-Gray) model estimates were obtained to ensure robustness of our findings.

1. *Do estimates vary depending on a IKNs reason for RAIHC assessment? Repeat multivariate models excluding those whose reason for assessment is a return from hospital or significant change in health status*
2. *Do estimates vary depending on the level of cognitive impairment one has? Repeat multivariable models excluding those whose CPS score is >=4*
3. *Does coefficient for COC or effect modification estimates vary depending on whether we restrict COC to individuals with 3 or more physician visits? In Sensitivity analyses, measure COC among individuals that have 3+ visits. see: Elizabeth Bayliss et al (2015) Effect of continuity of care on hospital utilization for seniors with multiple chronic conditions in an integrated health care system. Ann Fam Med; 13: 123-129*
4. *COC as tertiles (low, medium, high)*

JAN 2017 – at the request of the reviewers, construct a Wald test to confirm whether the subHR associated with low COC is the same for each level of multimorbidity (i.e., perform a Wald test to determine if the 4 interaction terms in the multivariable model are all equal to zero).

Appendix One:

Summary of Multimorbidity cohort

Prevalence of any one of 17 chronic diseases as of RAI-HC r1c

Conditions of interest:

1. acute myocardial infarction (AMI)
2. congestive heart failure (CHF)
3. asthma
4. chronic obstructive pulmonary disorder (COPD)
5. diabetes
6. hypertension
7. cardiac arrhythmia
8. chronic coronary syndrome
9. stroke (excluding transient ischemic attack)
10. osteoporosis
11. rheumatoid arthritis
12. osteo- and other arthritis
13. mood disorder
14. renal failure
15. all cancers
16. dementia*
17. mental illness other than dementia or depression

Entry into one of the 17 disease cohorts occurs by one or more of the following:

1. Present in ICES cohort: COPD, CHF, ODD, Asthma, or Hypertension with diagnosis date prior to index date
2. First AMI present in OMID in one year prior to index date
3. For conditions: rheumatoid arthritis, osteoarthritis, cardiac arrhythmia, dementia, osteoporosis, renal failure, stroke, or coronary syndrome
    - 1 acute care diagnosis present on admission (CIHI-DAD) in the period April 1, 2003-index
    - 2 OHIP office visit diagnoses within two years, with the first of the two visit dates defined as the diagnosis date, in the period April 1, 2003 – index.
4. For cancer, same as #3 above but use period April 1, 2010 – index (max 2 year lookback)
5. For dementia, use any CIHI, any ChEI (from ODB), or 3 OHIP office visits
6. For mental illness condition:
    - Acute care diagnosis present on admission (CIHI-DAD) using a 2 year lookback
    - OHIP office visit diagnoses within two years of index, with the first of the two visit dates defined as the diagnosis date

- OMHRS admissions in the period of Oct 1, 2005 – index (OMHRS collected starting from Oct 1, 2005). *Change: Consistent with previous HSPRN-multimorbidity work, OMHRS was not used for case ascertainment*

Diagnosis date for a chronic condition is defined as the earliest of the OHIP or DAD diagnosis dates (or CHEI) above, and must occur before the index date for inclusion.
